# Isolation and Structural Characterization of Eightfold Protonated Octacyanometalates [M(CNH)_8_]^4+^ (M=Mo^IV^, W^IV^) from Superacids

**DOI:** 10.1002/anie.202002366

**Published:** 2020-04-14

**Authors:** Malte Sellin, Valérie Marvaud, Moritz Malischewski

**Affiliations:** ^1^ Freie Universität Berlin Institut für Chemie und Biochemie Anorganische Chemie Fabeckstrasse 34–36 14195 Berlin Germany; ^2^ Sorbonne Université IPCM-CNRS-UMR8232, cc 229 4 place Jussieu 75252 Paris Cedex 05 France

**Keywords:** cyanides, hydrogen bonds, isocyanide ligands, protonation, superacidic systems

## Abstract

Octacyanometalates K_4_[Mo(CN)_8_] and K_4_[W(CN)_8_] are completely protonated in superacidic mixtures of anhydrous hydrogen fluoride and antimony pentafluoride. The resulting hydrogen isocyanide complexes [Mo(CNH)_8_]^4+^ [SbF_6_]^−^_4_ and [W(CNH)_8_]^4+^ [SbF_6_]^−^_4_ are the first examples of eight‐coordinate homoleptic metal complexes containing hydrogen isocyanide (CNH) ligands. The complexes were crystallographically characterized, revealing hydrogen‐bonded networks with short N⋅⋅⋅H⋅⋅⋅F contacts. Low‐temperature NMR measurements in HF confirmed rapid proton exchange even at −40 °C. Upon protonation, ν(C≡N) increases of about 50 cm^−1^ which is in agreement with DFT calculations.

## Graphical abstract

**Give me eight**! All eight cyano groups in the octacyanometalates Mo(CN)_8_^4−^ and W(CN)_8_^4−^ are protonated under superacidic conditions without formation of HCN. Instead homoleptic complexes of Mo^4+^ and W^4+^ with eight hydrogen isocyanide ligands are formed and could be fully characterized.

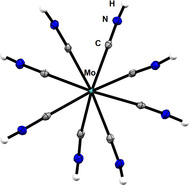

The lability of metal cyanides towards acids is well known and often a subject of safety warnings since highly toxic hydrogen cyanide might be released. In general, protonation of metal‐bound cyano ligands (M−C≡N) at the terminal nitrogen atom leads to the corresponding metal complex with hydrogen isocyanide as a ligand (M−C≡N−H)^+^.^1^, ^2^ In contrast, only a small number of metal complexes with hydrogen cyanide as a ligand are known (M−N≡C−H).^3^, ^4^, ^5^, ^6^, ^7^, ^8^, ^9^ Although hydrogen isocyanide CNH is a good ligand for transition metals, it can be displaced by donor solvents (e.g. water) or nucleophilic counteranions.^10^ Subsequently the liberated hydrogen isocyanide CNH can isomerize to its thermodynamically more stable tautomer, hydrogen cyanide HCN.

The superacidic mixtures HF/AsF_5_ or HF/SbF_5_ have recently been used for the protonation of organic nitriles^11^, ^12^ and even HCN^13^ as well as for the preparation of highly electrophilic organic cations.^14^, ^15^ Even though one could expect that the use of superacids should immediately lead to the destruction of polycyanometalates, these systems have the advantage that even the formed AsF_6_^−^ or SbF_6_^−^ anions are very weak nucleophiles and therefore much weaker ligands than the CNH ligands that are formed upon protonation.

Although the first reports on octacyanometalates [M(CN)_8_]^4−^ (M=Mo^IV^, W^IV^) date to the beginning of the 20th century,^16^, ^17^, ^18^, ^19^ they got a lot of attention from coordination and magnetochemists in the past decades. Since the early 2000s, a plethora of octacyanometalate‐based supramolecular coordination networks as well as polynuclear complexes and cluster compounds^20^, ^21^, ^22^, ^23^, ^24^ have been reported. The ease of oxidation of [M(CN)_8_]^4−^ (M=Mo, W) to [M(CN)_8_]^3−^ and the accessibility of an excited triplet state for [M(CN)_8_]^4−^ (M=Mo, W) by light irradiation make octacyanometalates suitable building blocks for photomagnetic materials,^25^, ^26^, ^27^, ^28^ while paramagnetic [M(CN)_8_]^3−^ (M=Mo, W) are promising building blocks for single‐molecule magnets.^29^

While treatment of octacyanometalates with hydrogen chloride gives adducts of the neutral acids H_4_[M(CN)_8_]⋅6 H_2_O (M=Mo, W),^30^ H_4_[W(CN)_8_]⋅4 HCl⋅12 H_2_O,^31^ and H_4_[Mo(CN)_8_]⋅2 O(C_2_H_5_)_2_⋅CH_3_OH⋅2 H_2_O,^32^ complete (octa‐) protonation is achieved by reacting K_4_[M(CN)_8_]⋅2 H_2_O (M=Mo, W) with anhydrous hydrogen fluoride and a large excess of antimony pentafluoride SbF_5_ (Scheme 1). Although the fully protonated species [M(CNH)_8_] [SbF_6_]_4_ (M=Mo, W) are only slightly soluble in anhydrous hydrogen fluoride at room temperature, their solubility can be slightly increased by adding small amounts of sulfur dioxide SO_2_ as cosolvent. Highly moisture‐sensitive yellow crystals form upon slow cooling to −75 °C besides colorless crystals of KSbF_6_.

**Scheme 1:**
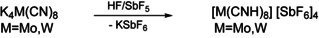
Preparation of [M(CNH)_8_]^4+^ [SbF_6_]^−^_4_ (M=Mo, W).

[Mo(CNH)_8_]^4+^ [SbF_6_]^−^_4_⋅2 HF and [W(CNH)_8_]^4+^ [SbF_6_]^−^_4_⋅2 HF both crystallize in the monoclinic space group *P*2_1_/*n* and are isomorphous. The central metal is coordinated by eight (crystallographically different) protonated cyanide/hydrogen isocyanide ligands (M−CNH), resulting in a slightly distorted square‐antiprismatic coordination geometry. The question whether the ligands are coordinated to the metal via carbon (M−C≡NH) or nitrogen (M−N≡CH) could be clearly answered by comparing the R factors and atomic displacement parameters of both structure solutions (see the Supporting Information). Due to the high overall data quality the positions of all hydrogen atoms could be located via difference electron density map.

The M−C−N−H bonds are close to linear (varying from 172–179°; Figure 1), while all terminal hydrogen atoms of the hydrogen isocyanide ligands form strong hydrogen bonds to the fluorine atoms of the SbF_6_^−^ ions and cocrystallized HF molecules. In the molybdenum compound the MCN**H**⋅⋅⋅**F** distances are in the range of 1.718(3)–1.994(4) Å. Thus, the **N**−(H)⋅⋅⋅**F** distances are relatively short (2.584(2)–2.709(2) Å) and in a similar range to those in protonated nitriles with hexafluorometalate counteranions (2.5–2.8 Å).^11^, ^12^, ^13^

**Figure 1:**
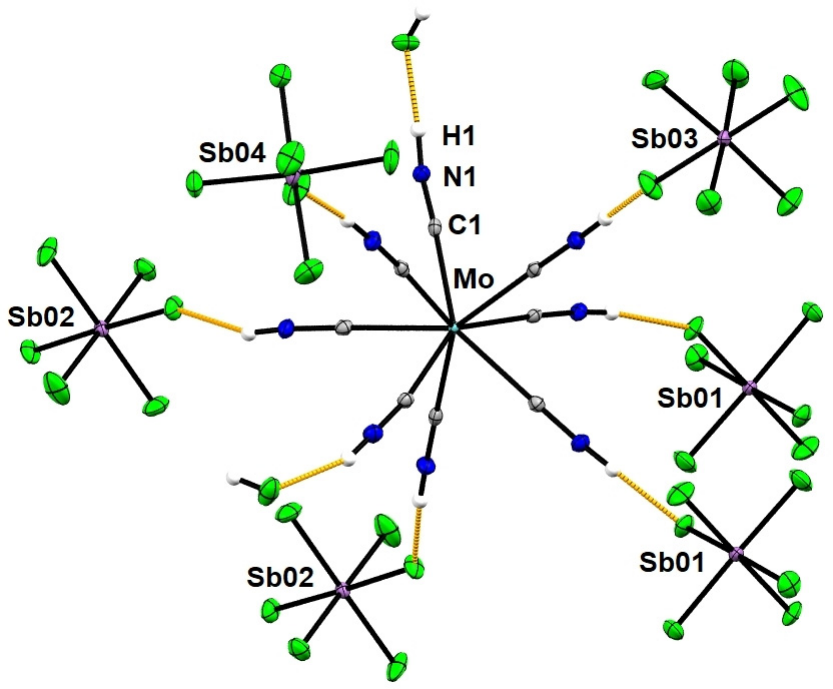
Selected short H⋅⋅⋅F contacts <2 Å (in orange) in the crystal structure of [Mo(CNH)_8_]^4+^ [SbF_6_]^−^_4_⋅2 HF. Ellipsoids shown at 50 % probability; C gray, N blue, H white, Mo turquoise, F green, Sb lavender.

The Mo−C bond lengths in [Mo(CNH)_8_]^4+^ [SbF_6_]^−^_4_⋅2 HF (2.140(2)–2.168(2) Å) are very similar to the Mo−C bond lengths in K_4_[Mo(CN)_8_]⋅2 H_2_O (Mo−C 2.163(5) Å).^33^ However, changes in C−N bond lengths are more significant. In the fully protonated species, the C−N bond lengths are in the range of 1.128(3)–1.136(3) Å and therefore shorter than in the potassium salt (1.152(6) Å). While the former value resembles more the C−N bond length in protonated nitriles,^11^ the latter is more similar to free HCN (1.157(1) Å).^34^ Bond lengths in [W(CNH)_8_]^4+^ [SbF_6_]^−^_4_⋅2 HF are very similar to the analogous Mo compound (see Table 1). This finding is also supported by DFT calculations (M06L/Def2TZVP) on both [M(CNH)_8_]^4+^ and [M(CNH)_8_]^4+^⋅8 HF (M=Mo, W). The latter was chosen as a model to simulate the influence of hydrogen bonding in the crystal. Interestingly, the comparison revealed that C−N bond lengths were totally unaffected, while M−C bond lengths decreased slightly in the calculated HF solvates. However, it has to be stated that the calculated M−C bond lengths were significantly longer than the experimentally found values.

**Table 1:** Experimental and calculated bond lengths in Å.

Compound	M−C (expt.).	C−N (expt.)	M−C (calc.)	C−N (calc.)
[Mo(CNH)_8_]^4+^	2.140(2)– 2.168(2)	1.128(3)– 1.136(3)	2.203	1.146
[W(CNH)_8_]^4+^	2.142(2)– 2.169(2)	1.127(3)– 1.137(3)	2.211	1.147

Additionally, frequency calculations turned out to be even more problematic. The comparison between the calculations for [M(CNH)_8_]^4+^, [M(NCH)_8_]^4+^, [M(CNH)_8_]^4+^⋅8 HF, and [M(NCH)_8_]^4+^⋅8 HF (M=Mo, W) with the reaction products was inconclusive. Probably this is caused by the high ionic charges and strong hydrogen bonding which are insufficiently modeled in the calculations.

The IR spectra of [M(CNH)_8_]^4+^ [SbF_6_]^−^_4_⋅2 HF (M=Mo, W; Figure 2) both display a very broad band above 3000 cm^−1^ which can be attributed to N–H stretching. Additionally, a weak band at 1615 cm^−1^ can be assigned to N–H bending, since both bands were shifted during deuteration experiments with DF/SbF_5_. While an isotopic ratio of 1.37 is observed for the δ(NH)/δ(ND) deformation vibrations (close to the theoretical value of 1.41) the corresponding value for the ν(NH)/ν(ND) stretching vibrations is only ≈1.2 (Table 2). Similar effects have been observed before^35^ and are caused by strong hydrogen bonding, which has a greater influence on stretching vibrations than on deformation vibrations.

**Figure 2:**
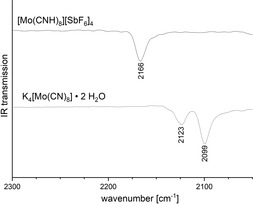
IR spectra showing the shifted CN stretching frequency upon protonation.

**Table 2:** Experimental IR data in cm^−1^.

	[Mo(CNH)_8_]^4+^	[Mo(CND)_8_]^4+^	[W(CNH)_8_]^4+^	[W(CND)_8_]^4+^
ν(NH)/ ν(ND)	3082 (b)	2529 (b)	3030 (b)	2525 (b)
ν(CN)	2166 (m)	1960 (b)	2145 (m)	1970 (b)
δ(NH)/ δ(ND)	1615 (m)	1182 (m)	1620 (m)	1180 (m)

IR and Raman spectra of [M(CNH)_8_]^4+^ [SbF_6_]^−^_4_⋅2 HF (M=Mo, W) both display an increase of the CN stretching vibration by about 50 cm^−1^ compared to K_4_[M(CN)_8_]⋅2 H_2_O. A similar blueshift has already been observed in IR spectra of neutral polycyanometalate acids.^36^, ^37^, ^38^, ^39^, ^40^ This bond‐strengthening effect upon protonation is caused by the increased polarization of the carbon–nitrogen bond. This observation is in line with the shortening of the carbon–nitrogen distance in the solid state structure.

Despite the relatively low solubility of [M(CNH)_8_]^4+^ [SbF_6_]^−^_4_ (M=Mo, W) in pure anhydrous HF even at room temperature, it was possible to record NMR spectra of the products by using a solvent mixture of HF and SO_2_ at −40 °C (Table 3). The ^14^N NMR spectrum of a solution of [Mo(CNH)_8_]^4+^ [SbF_6_]^−^_4_ shows a broad, unresolved peak at (*δ*=−182 ppm) which is significantly shifted compared to K_4_Mo(CN)_8_ in water (*δ*=−95 ppm). A similar shift was observed for the protonation of acetonitrile (*δ*(CH_3_CN=−134 ppm; *δ*(CH_3_CNH^+^)=−241 ppm).^11^ Only one signal at *δ*=121 ppm is displayed in the ^13^C NMR spectrum, which indicates an upfield shift upon protonation compared to aqueous K_4_(Mo(CN)_8_ (*δ*=149 ppm).

**Table 3:** NMR data, recorded in a mixture of HF and SO_2_ at −40°C; chemical shifts *δ* in ppm.

	^13^C	^14^N	
K_4_Mo(CN)_8_ in D_2_O	+149	−95	
K_4_W(CN)_8_ in D_2_O	+143	−98	
[Mo(CNH)_8_]^4+^ [SbF_6_]^−^_4_ in HF	+121	−182	
[W(CNH)_8_]^4+^ [SbF_6_]−_4_ in HF	+115	−179

It was not possible to detect a peak for the CNH ligand in the ^1^H NMR spectrum, since rapid exchange between the CNH group and the highly acidic solvent mixture is expected. A similar problem was reported for the protonation of H_4_Fe(CN)_6_ by HF/BF_3_ where it was not possible to freeze‐out proton exchange even at the melting point of the solvent (−84 °C).^41^ Although the product [Fe(CNH)_6_][BF_4_]_2_ was reported to be stable for months in anhydrous HF, it decomposed in vacuum by losing HF and BF_3_ to give H_4_Fe(CN)_6_. However, it has to be stated that under much more basic conditions, namely in presence of ethanol, a so‐called supramolecular complex with the formula [Fe{CNH−O(H)Et}_6_]Cl_2_ was crystallographically characterized.^42^

In summary, we report the first successful isolation of homoleptic metal complexes with eight hydrogen isocyanide ligands by exhaustive protonation of K_4_M(CN)_8_ by the superacid HF/SbF_5_. Since isocyanides CNR are good σ‐donor but weak π‐acceptor ligands,^43^ they provide an effective stabilization of the Mo^4+^ and W^4+^ ions. The resulting square‐antiprismatic complexes [M(CNH)_8_]^4+^ (M=Mo^IV^, W^IV^) are diamagnetic and fulfill the 18‐electron rule. While M−C bond lengths remain almost unchanged, protonation slightly shortens the C≡N bond, which is supported by an increase of ν(CN) by 50 cm^−1^. Additionally, the crystal structures display networks of strong H⋅⋅⋅F hydrogen bonds. These results suggest that polycyanometalates are much more stable against protolysis than generally thought (at least in the absence of potent nucleophiles) which opens up new pathways to hydrogen‐bonded networks for various applications.^44^

## Conflict of interest

The authors declare no conflict of interest.

## Supporting information

As a service to our authors and readers, this journal provides supporting information supplied by the authors. Such materials are peer reviewed and may be re‐organized for online delivery, but are not copy‐edited or typeset. Technical support issues arising from supporting information (other than missing files) should be addressed to the authors.

Supplementary
