# Supplementary material for: Isolation and Structural Characterization of Eightfold Protonated Octacyanometalates [M(CNH)8]4+ (M=MoIV, WIV) from Superacids
Source: Angew Chem Int Ed Engl. 2020 Apr 14;59(26):10519–22. doi: 10.1002/anie.202002366 (PMC7317926; doi:10.1002/anie.202002366)
Supplement: Supplementary file 1 — Supplementary [file ANIE-59-10519-s001.pdf]

Angewandte Chemie
Eine Zeitschrift der Gesellschaft Deutscher Chemiker

Supporting Information

Isolation and Structural Characterization of Eightfold Protonated Octacyanometalates $[M(CNH)_8]^{4+}$ ($M = Mo^{IV}$, W^{IV}) from Superacids

*Malte Sellin, Valérie Marvaud, and Moritz Malischewski**

anie_202002366_sm_miscellaneous_information.pdf

Content

1. Experimental Details 1
2. IR and Raman-Spectra 3
3. NMR-spectra 7
3.1 NMR-spectra of the educts 7
3.2 NMR-spectra of the products 9
4. Crystal Structures 11
5. Continuous Shape-Measurements 19
6. DFT-Calculations 24
7. References 32

1. Experimental Details

The reactions were performed in PFA (tetrafluoroethene-perfluoroalkoxyvinyl-copolymer) tubes with the help of a stainless steel vacuum line. Instead of stirring the mixtures were agitated with the help of the Mini-Vortex Mixer PV-1. SbF_5 and HF are highly dangerous compounds with devastating effects on human tissue. They should only be handled in appropriate equipment by trained personnel. Anhydrous HF and SO_2 were stored in stainless steel cyclinders. HF was distilled from K_2NiF_6 before use while SO_2 was stored over CaH_2. Deuterium Fluoride DF was purchased from ABCR.

$K_4[Mo(CN)_8] \bullet 2H_2O$[1] and $K_4[W(CN)_8] \bullet 2H_2O$[2] were prepared by literature procedures.

IR Spectra were recorded on a Bruker ALPHA FTIR spectrometer inside a glovebox equipped with a diamond ATR attachment. Raman Spectra were measured with a Bruker RamanScope III. NMR Spectra of the superacidic solutions were measured in sealed 5mm PFA tubes inside 6mm NMR glass tubes containing d^6-acetone on a JEOL 400 MHz ECZ spectrometer. Single-crystals were decanted off the cold $HF/SO_2/SbF_5$ solution (Danger!) and manipulated in a cold nitrogen stream.

Preparation of $[W(CNH)_8]^{4+}$ $[SbF_6]^-_4 \bullet 2HF$:

Inside a glovebox, antimony pentafluoride (200 mg, 0.922 mmol) was filled into a 8 mm PFA tube equipped with a stainless steel valve. Anhydrous HF (0.8 mL) was condensed in at -196°C. Subsequently, potassium octacyanotungstate(IV) dihydrate (30 mg, 0.055 mmol) was added to the frozen solution. The mixture was brought to room temperature, resulting in a yellow solution with a yellow precipitate at room temperature. To increase the solubility sulfur dioxide (0.2 mL) was condensed into the mixture. This resulted in a clear yellow solution at room temperature. The mixture was slowly cooled to -78°C in a freezer. The next day, moisture-sensitive yellow crystals of $[W(CNH)_8]^{4+}$ $[SbF_6]^-_4 \bullet 2HF$ had formed in the almost colourless solution besides colorless crystals of $KSbF_6$ and H_3OSbF_6.

$[W(CNH)_8][SbF_6]_4$

^1H NMR (400.5 MHz, HF/SO_2, ext.[d^6]acetone, -40 °C): no signal observed (fast exchange with solvent)

$^{13}C\{^1H\}$ NMR (100.7 MHz, HF/SO_2, ext.[d^6]acetone, - 40°C): +115.4 ppm

^{14}N NMR (28.9 MHz, HF/SO_2, ext.[d^6]acetone, -40 °C): -178.6 ppm (broad)

^{19}F NMR (369.7 MHz, HF/SO_2, ext.[d^6]acetone, -40°C): -120 ppm (very broad)

ṽ (IR, ATR powder) = 3030 b, 2145 m, 1620 m, 773 m, 661 sh, 634 vs, 569 s, 473 m cm^{-1}

ṽ (raman, crystal) = 2183 vs, 2176 s, 666 s, 639 m, 580 w, 547 w, 463 w, 280 m, 162 m cm^{-1}

$[W(CND)_8][SbF_6]_4$

ṽ (IR, ATR powder) = 2525 b, 2161 w, 1970 b, 1180 m, 677 sh, 652 vs, 485 s cm^{-1}

The reaction was performed accordingly, but DF instead of HF was used.

Preparation of $[Mo(CNH)_8]^{4+}$ $[SbF_6]^-_4$ • 2HF:

The same procedure was performed with potassium octacyanomolybdate(IV) dihydrate (24 mg, 0.052 mmol).

$[Mo(CNH)_8][SbF_6]_4$

^1H NMR (400.5 MHz, HF/SO_2, ext.[d^6]acetone, -40 °C): no signal observed (fast exchange with solvent)

$^{13}C\{^1H\}$ NMR (100.7 MHz, HF/SO_2, ext.[d^6]acetone, - 40°C): +121.1 ppm

^{14}N NMR (28.9 MHz, HF/SO_2, ext.[d^6]acetone, -40 °C): -181.8 ppm (broad)

^{19}F NMR (369.7 MHz, HF/SO_2, ext.[d^6]acetone, -40°C): -120 ppm (very broad)

ṽ (IR, ATR powder) = 3082 b, 2166 m, 1615 m, 677 sh, 655 vs, 591 s, 487 s cm^{-1}

ṽ (raman, crystal) = 2191 s, 2174 vs, 666 sh, 650 s, 575 w, 543 w, 457 w, 286 m, 168 sh, 153 s cm^{-1}

$[Mo(CND)_8][SbF_6]_4$

The reaction was performed accordingly, but DF instead of HF was used.

ṽ (IR, ATR powder) = 2529 b, 2166 w, 1960 b, 1182 m, 679 sh, 655 vs, 485 s cm^{-1}

2. IR and Raman-Spectra

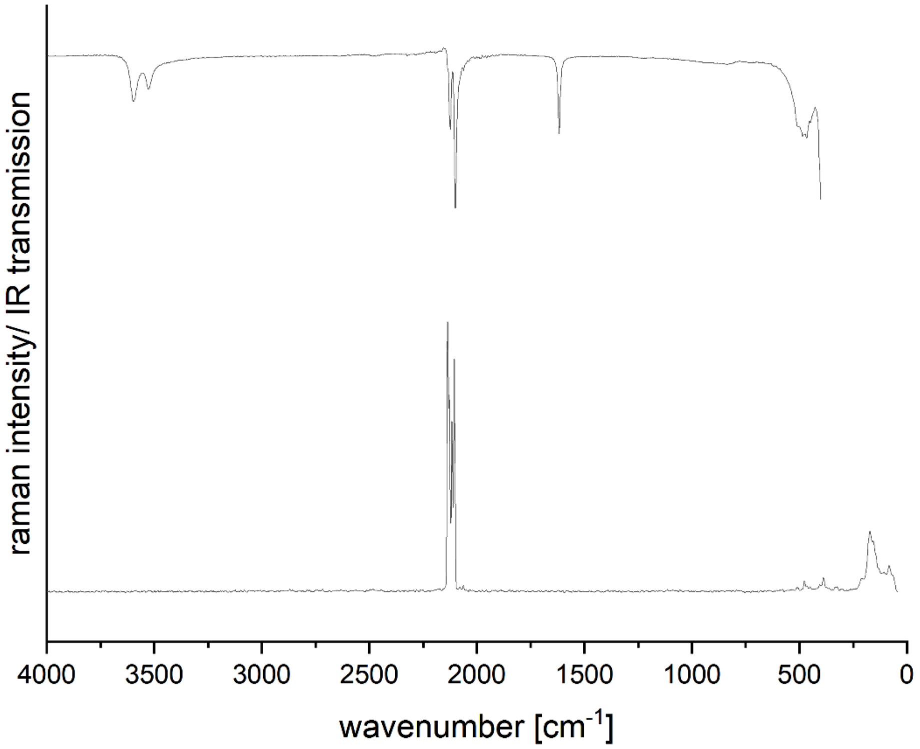

Figure S1. IR/raman spectrum of $K_4[Mo(CN)_8] \bullet 2H_2O$ at room temperature.

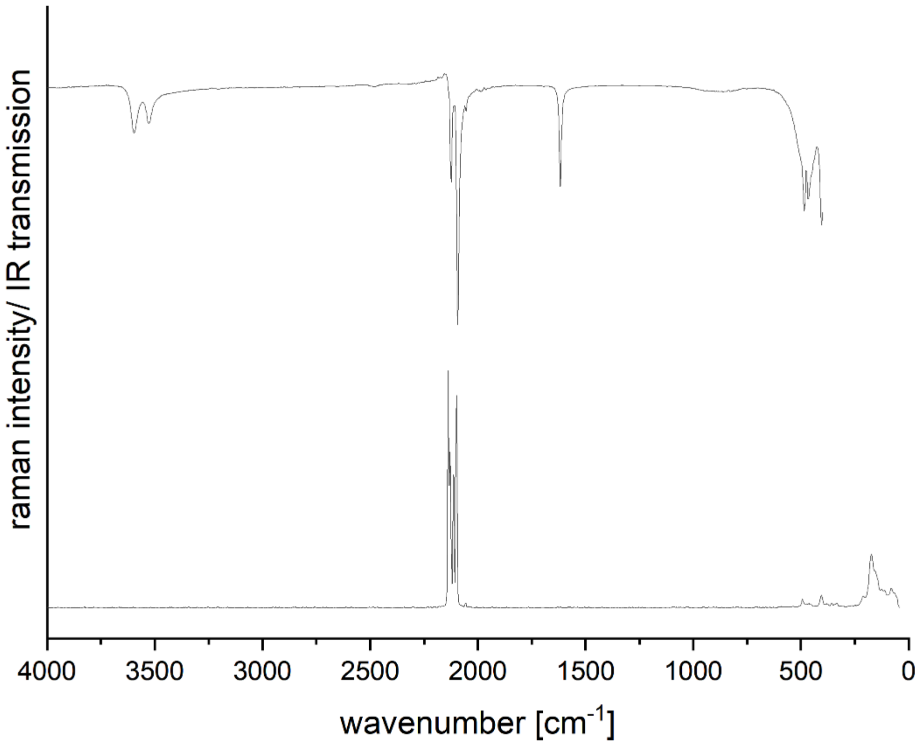

Figure S2. IR/raman spectrum of $K_4[W(CN)_8] \bullet 2H_2O$ at room temperature.

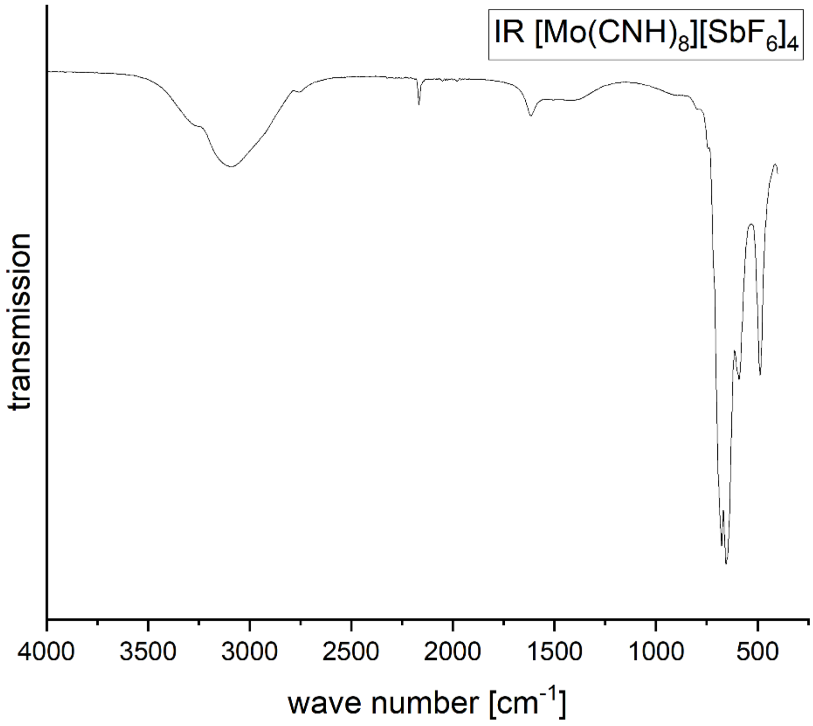

Figure S3. IR spectrum of $[Mo(CNH)_8][SbF_6]_4$

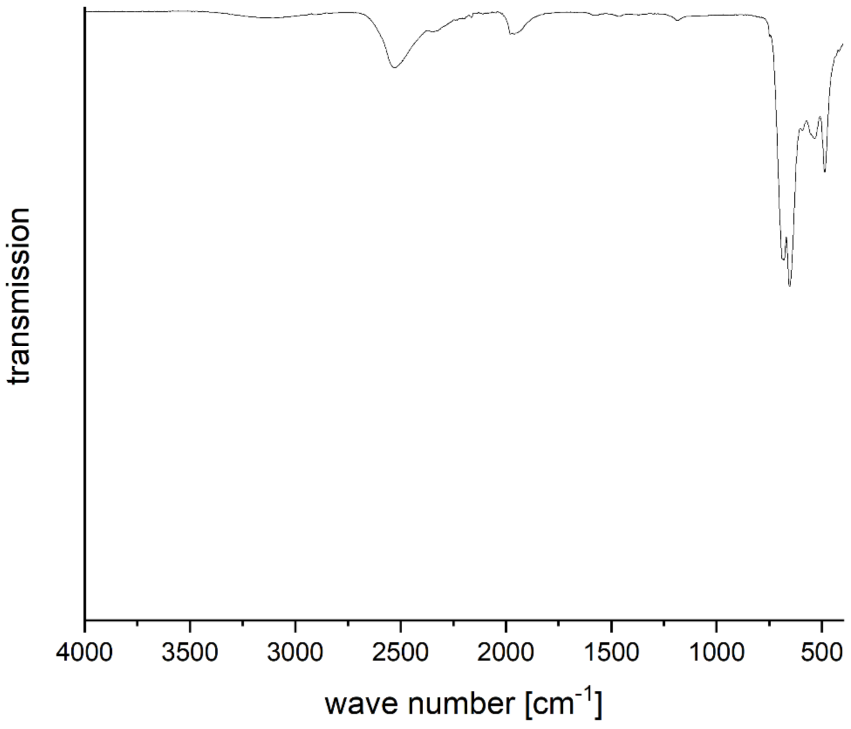

Figure S3. IR spectrum of $[Mo(CND)_8][SbF_6]_4$

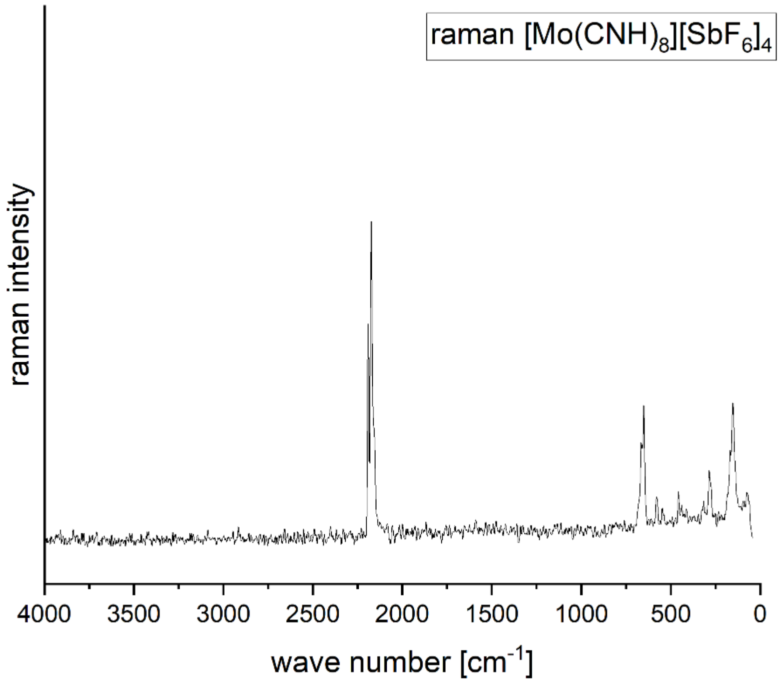

Figure S5. Raman spectrum of $[Mo(CNH)_8][SbF_6]_4$

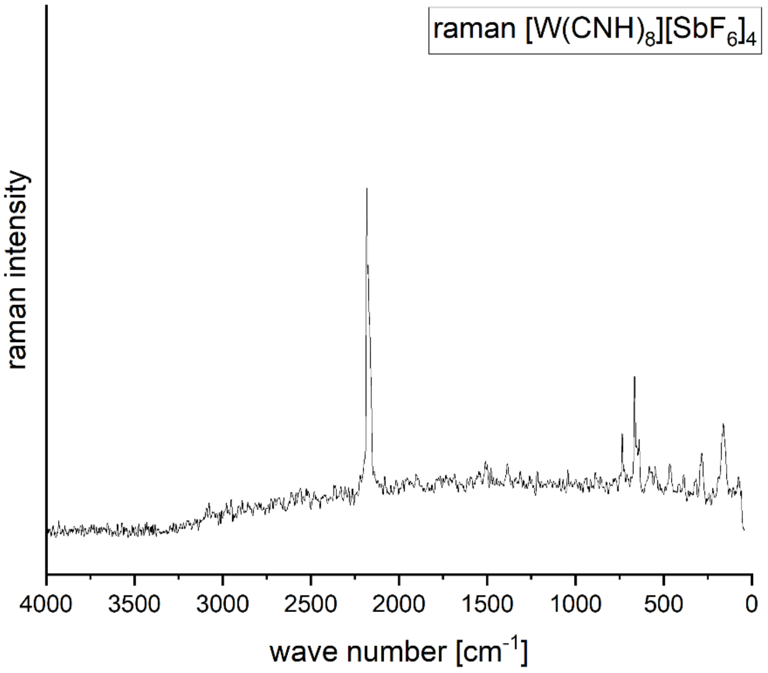

Figure S6. Raman spectrum of $[W(CNH)_8][SbF_6]_4$

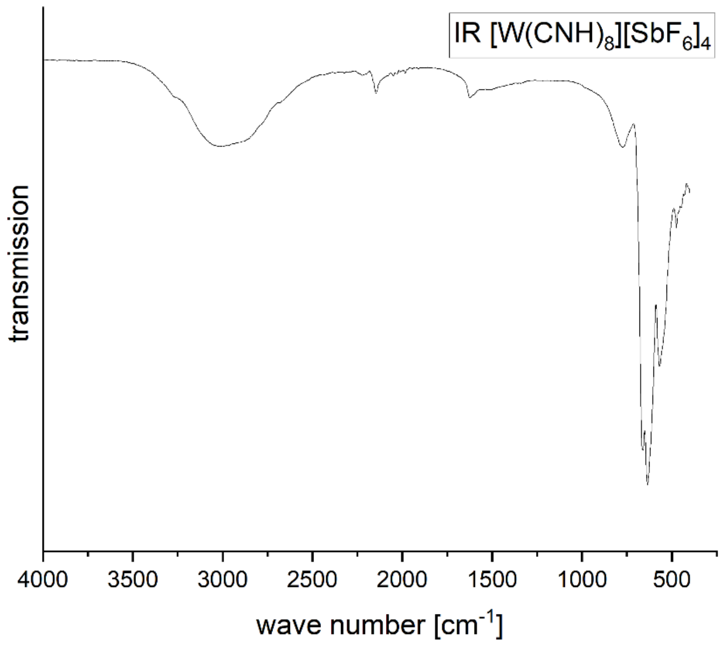

Figure S6. IR Spectrum of [W(CNH)$_8$][SbF$_6$]$_4$

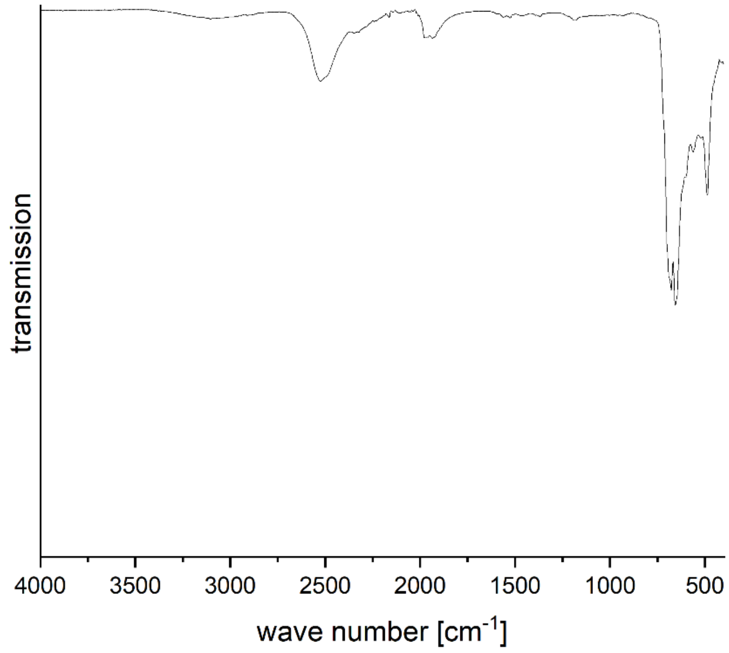

Figure S7. IR Spectrum of [W(CND)$_8$][SbF$_6$]$_4$

3. NMR-spectra

3.1 NMR-spectra of the educts

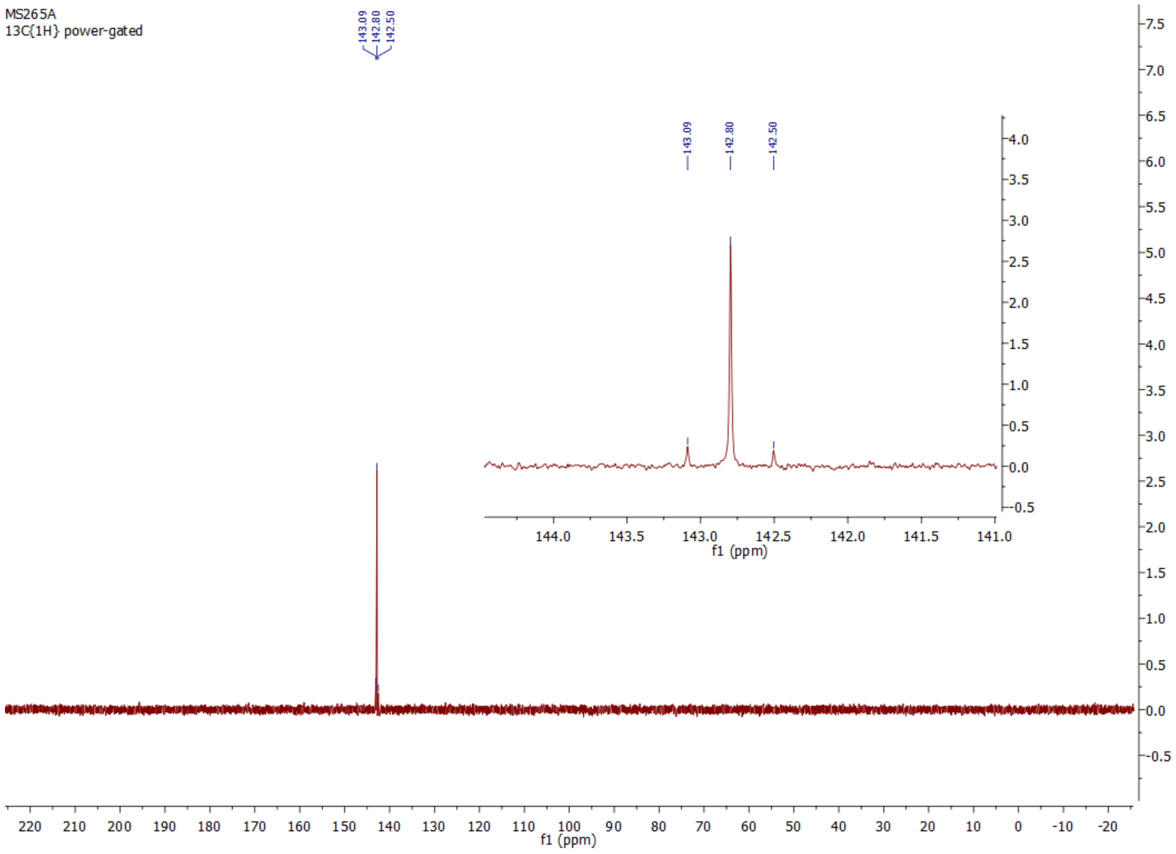

Figure S8. ^{13}C NMR Spectrum of $K_4W(CN)_8$ in D_2O at room temperature

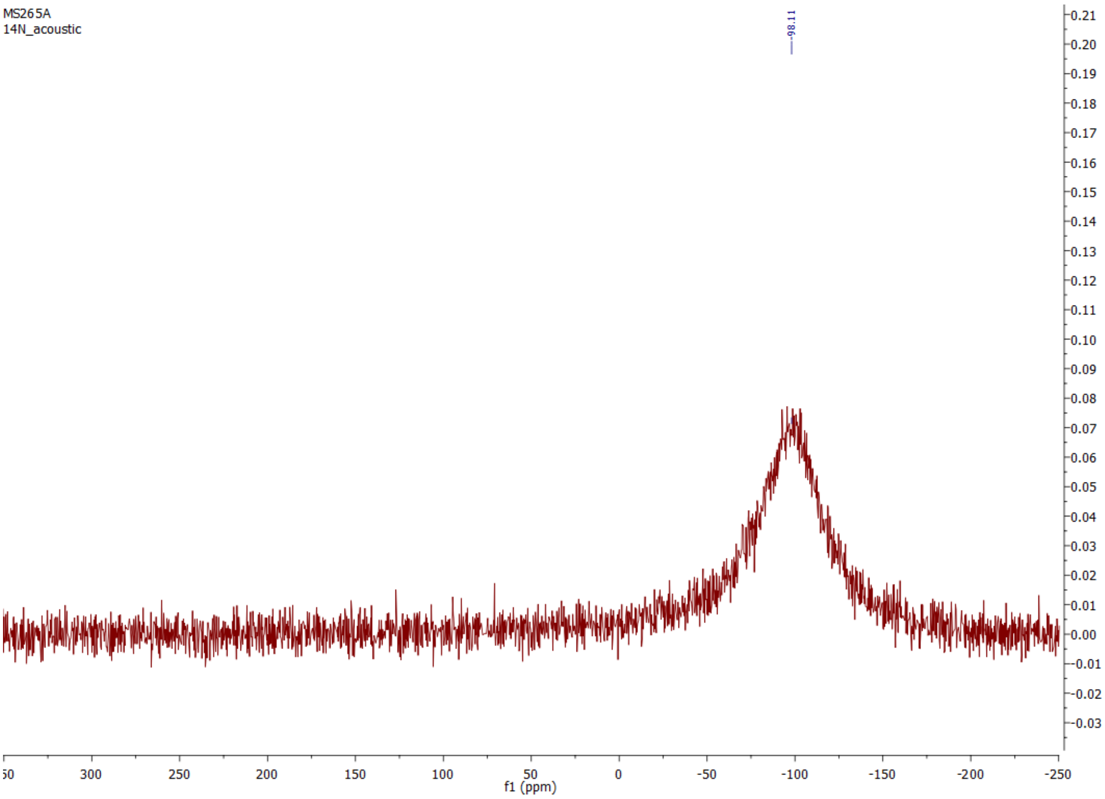

Figure S9. ^{14}N NMR spectrum of $K_4W(CN)_8$ in D_2O at room temperature

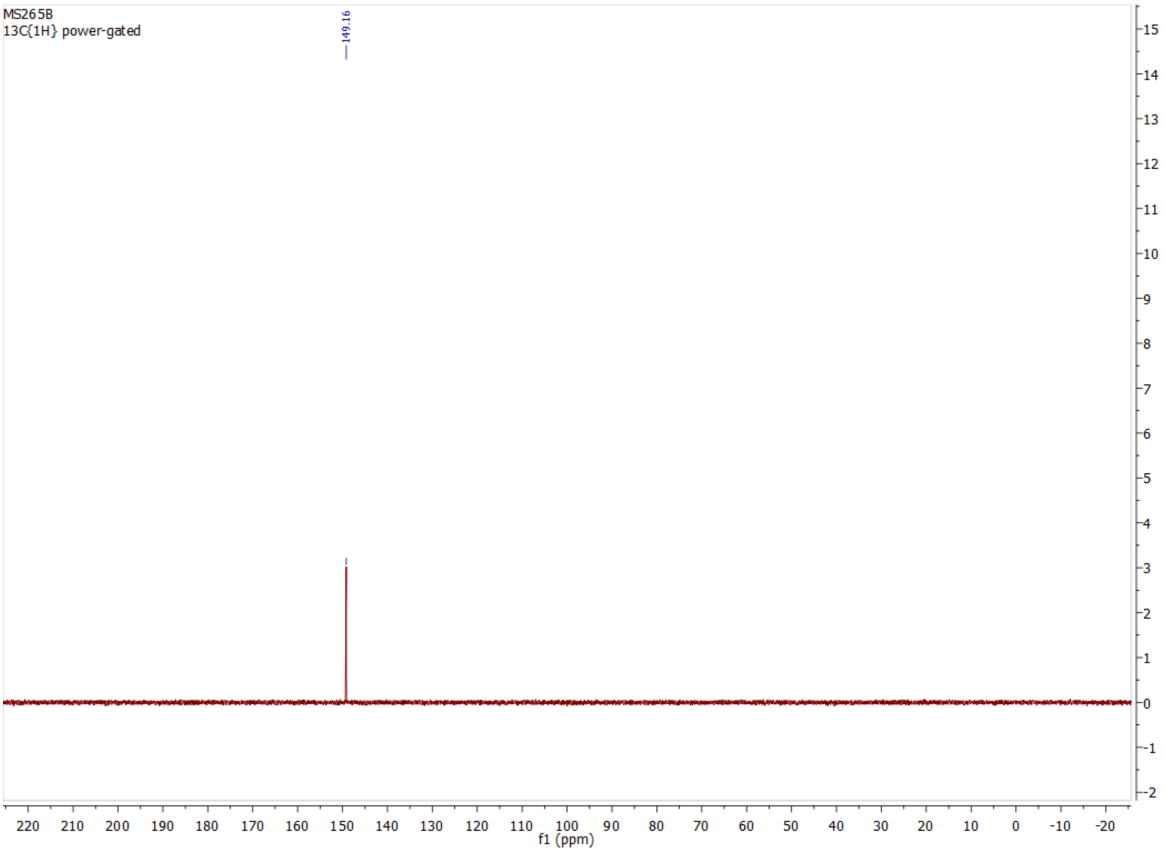

Figure S10. ^{13}C NMR Spectrum of $K_4Mo(CN)_8$ in D_2O at room temperature

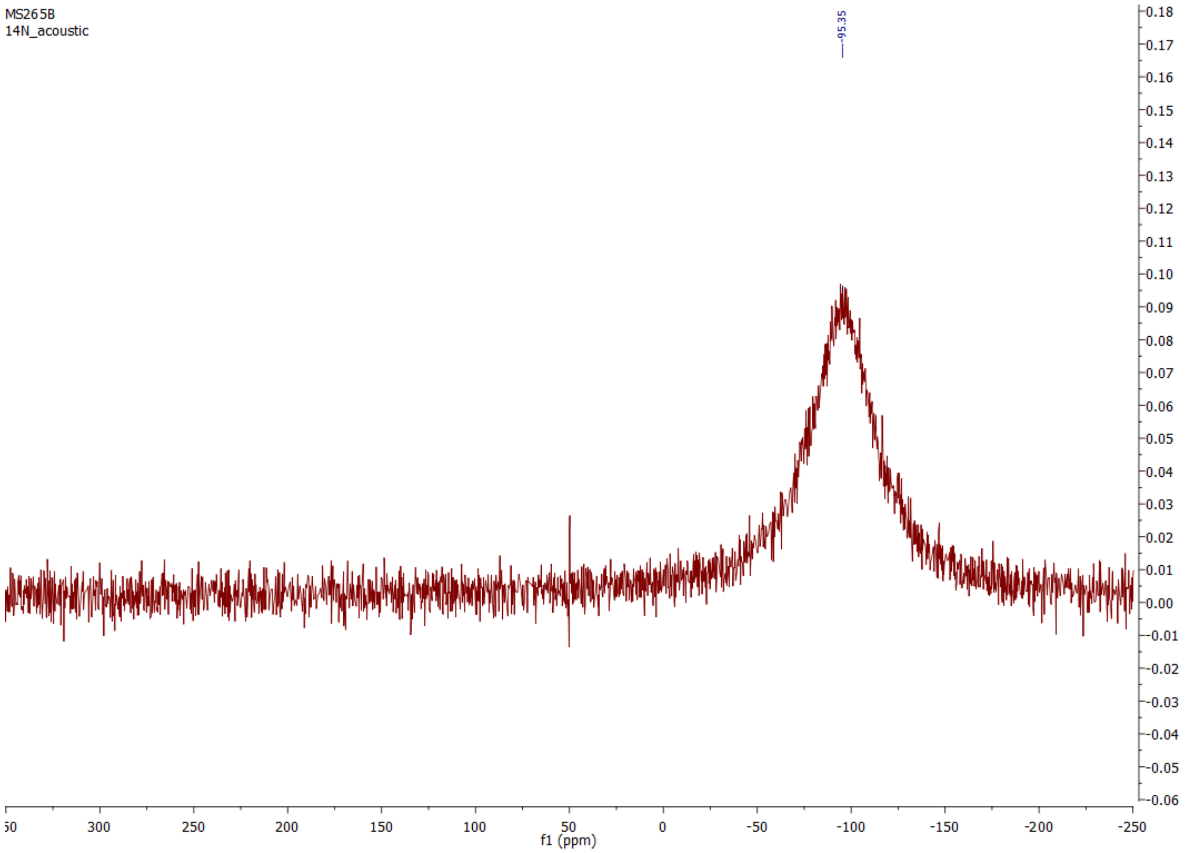

Figure S11. ^{14}N NMR Spectrum of $K_4Mo(CN)_8$ in D_2O at room temperature

3.2 NMR-spectra of the products

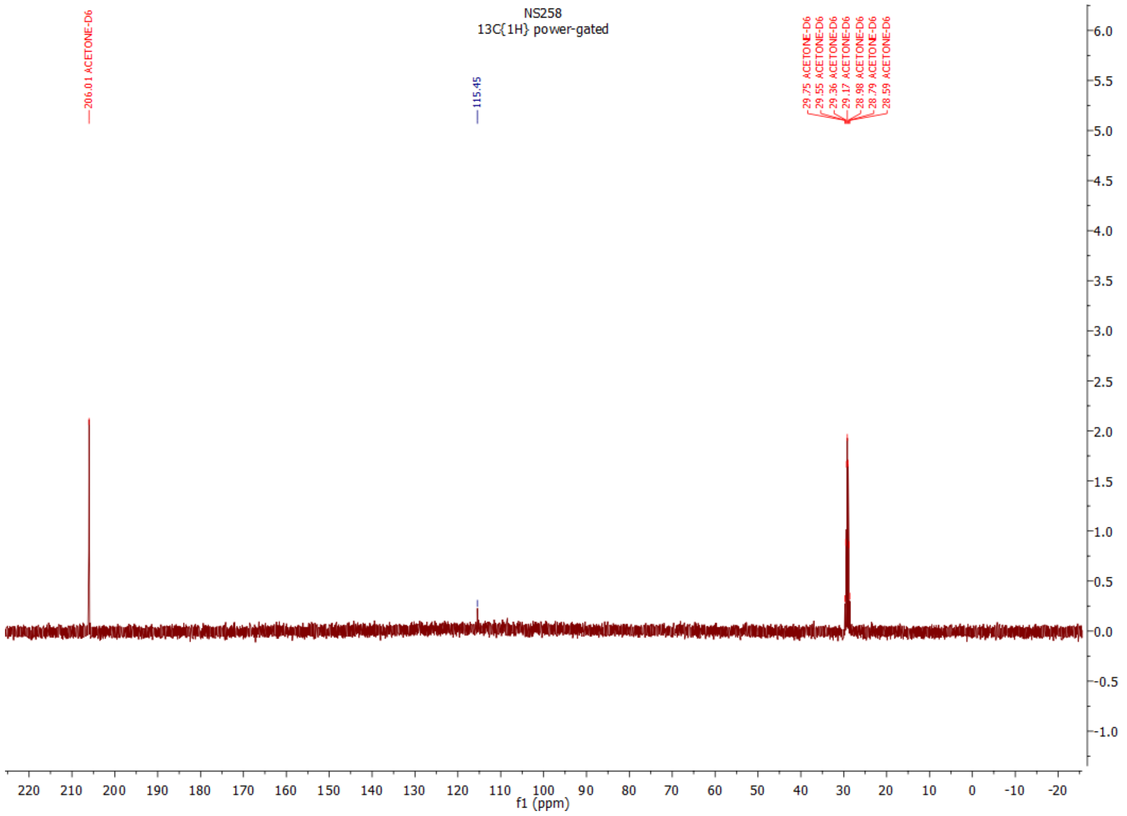

Figure S12. ^{13}C NMR Spectrum of $[W(CNH)_8]^{4+}$ $[SbF_6]^-_4$ in HF – ext. reference d^6-acetone

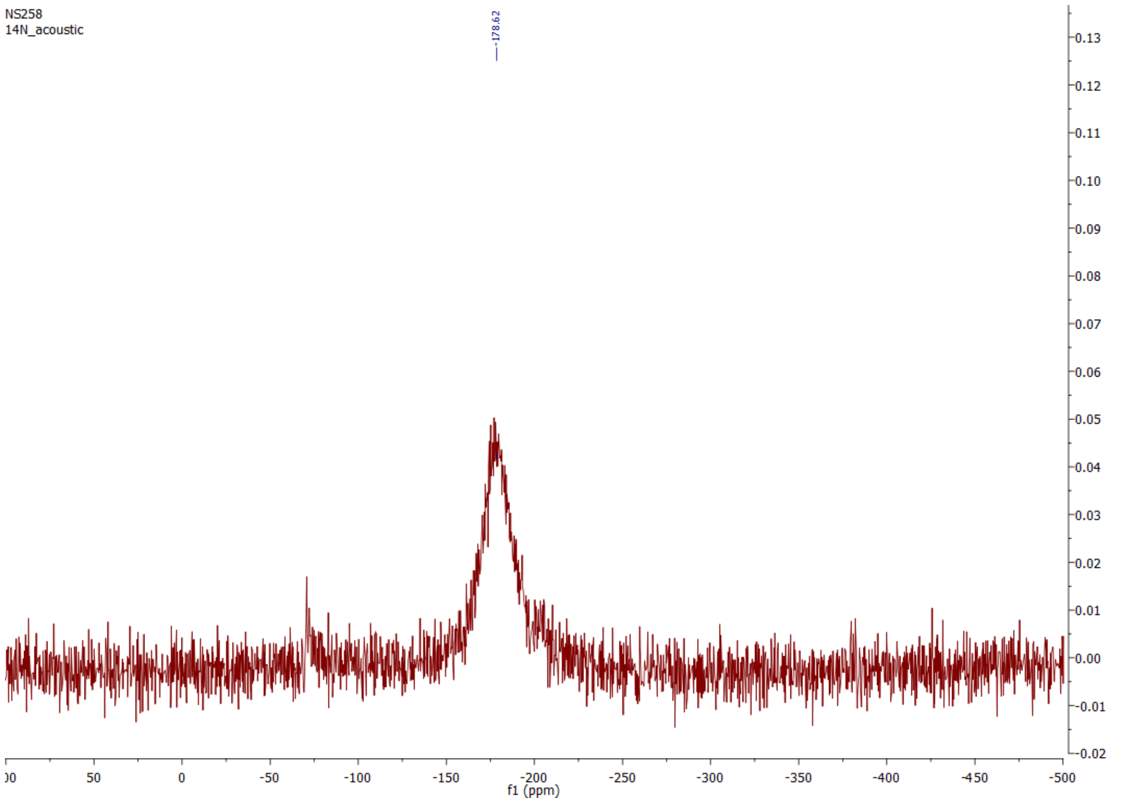

Figure S12. ^{14}N NMR Spectrum of $[W(CNH)_8]^{4+}$ $[SbF_6]^-_4$ in HF – ext. reference d^6-acetone

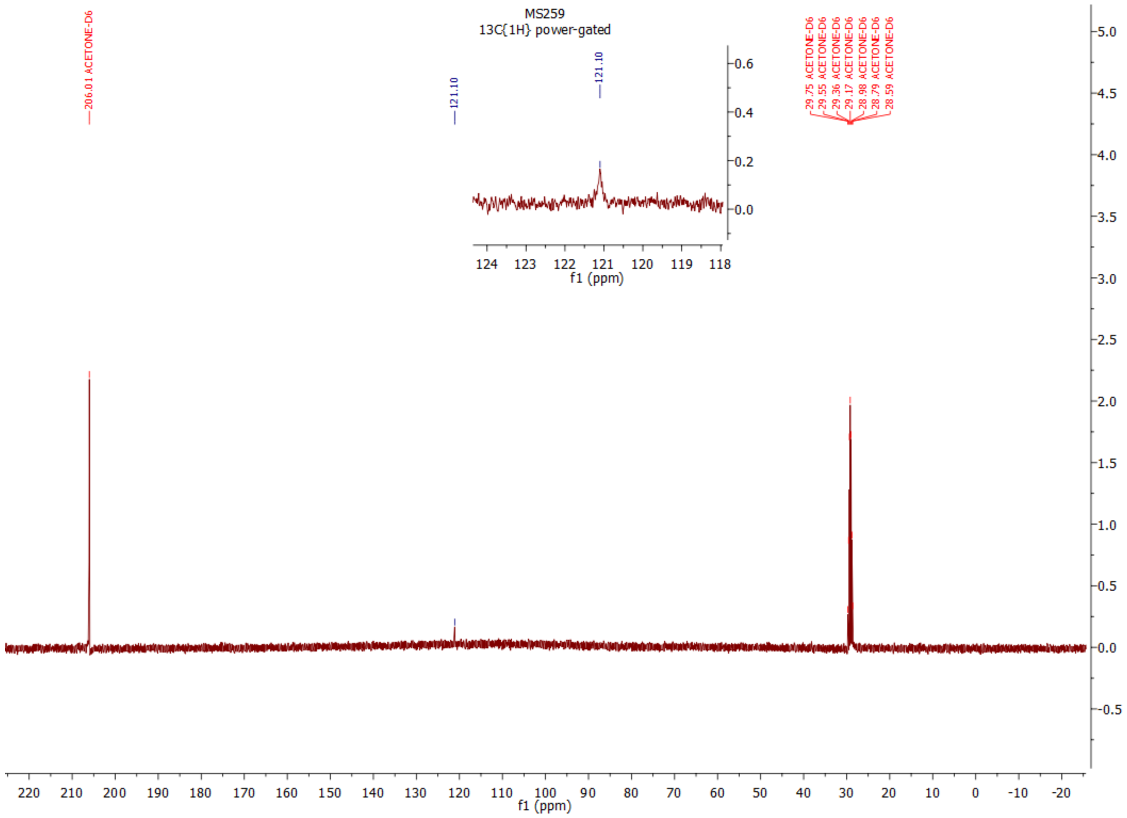

Figure S13. ^{13}C NMR of $[Mo(CNH)_8]^{4+}$ $[SbF_6]^-_4$ in HF– ext. reference d^6-acetone

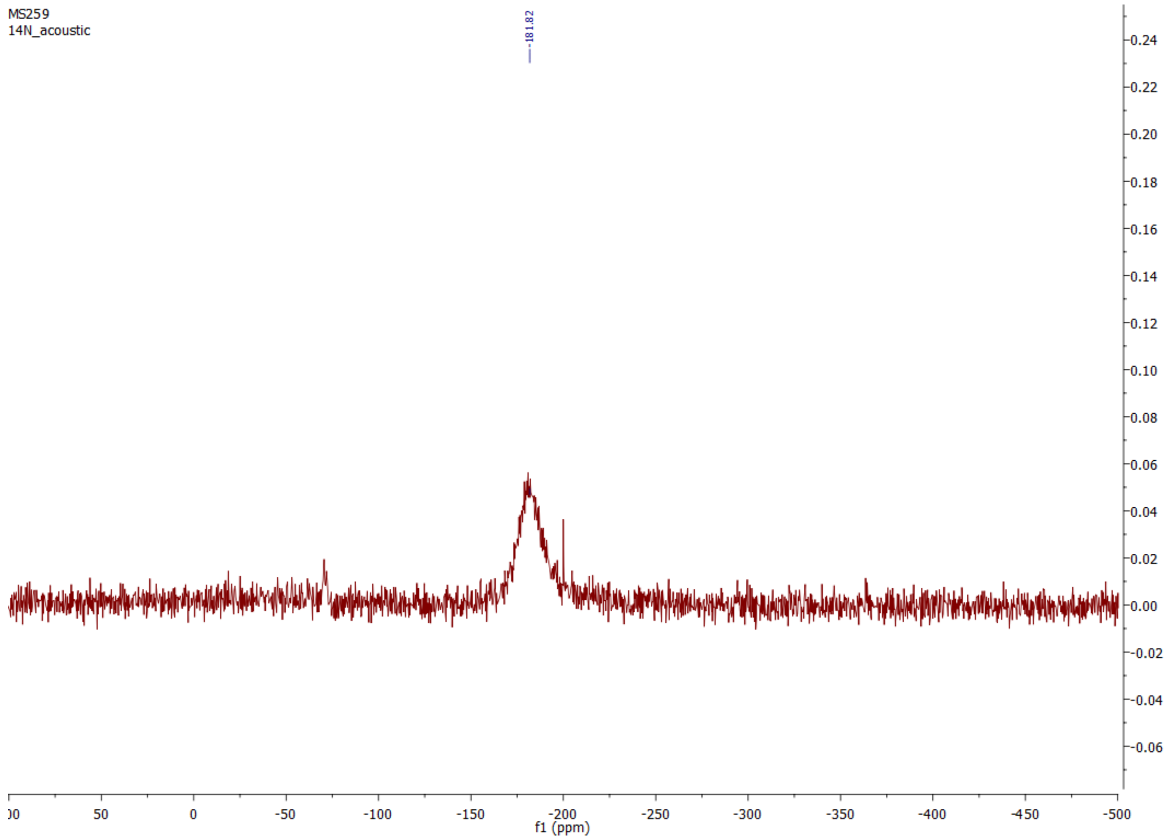

Figure S14. ^{14}N NMR of $[Mo(CNH)_8]^{4+}$ $[SbF_6]^-_4$ in HF– ext. reference d^6-acetone

4. Crystal Structures

Single crystals were mounted in a cold nitrogen stream on a Bruker D8 Venture diffractometer using Mo Kα-radiation. X-ray structures were solved and refined using SHELXL [3], software from Bruker[4] and OLEX.[5] Drawings and tables were generated with Mercury[6] and publCIF.[7]

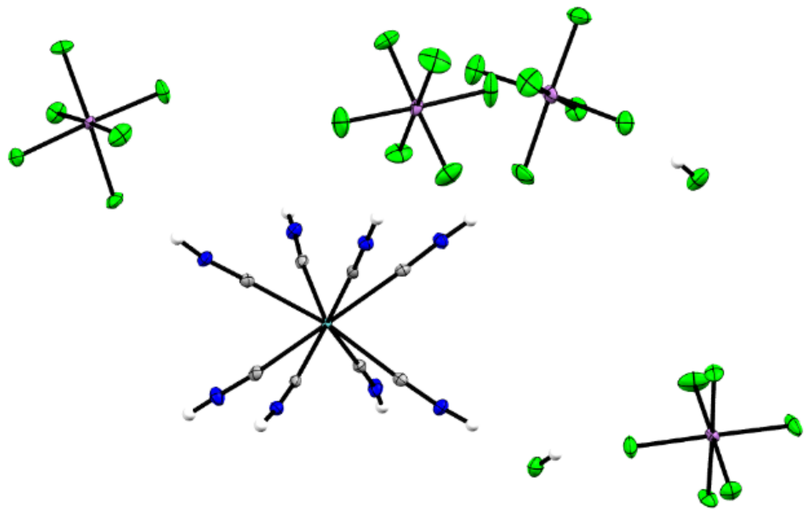

Figure S15. One formula unit in the crystal structure of $[Mo(CNH)_8]^{4+}$ $[SbF_6]^-_4$ • 2HF , ellipsoids shown at 50% probability, C grey, N blue, H white, Mo turquoise, F green, Sb lavender

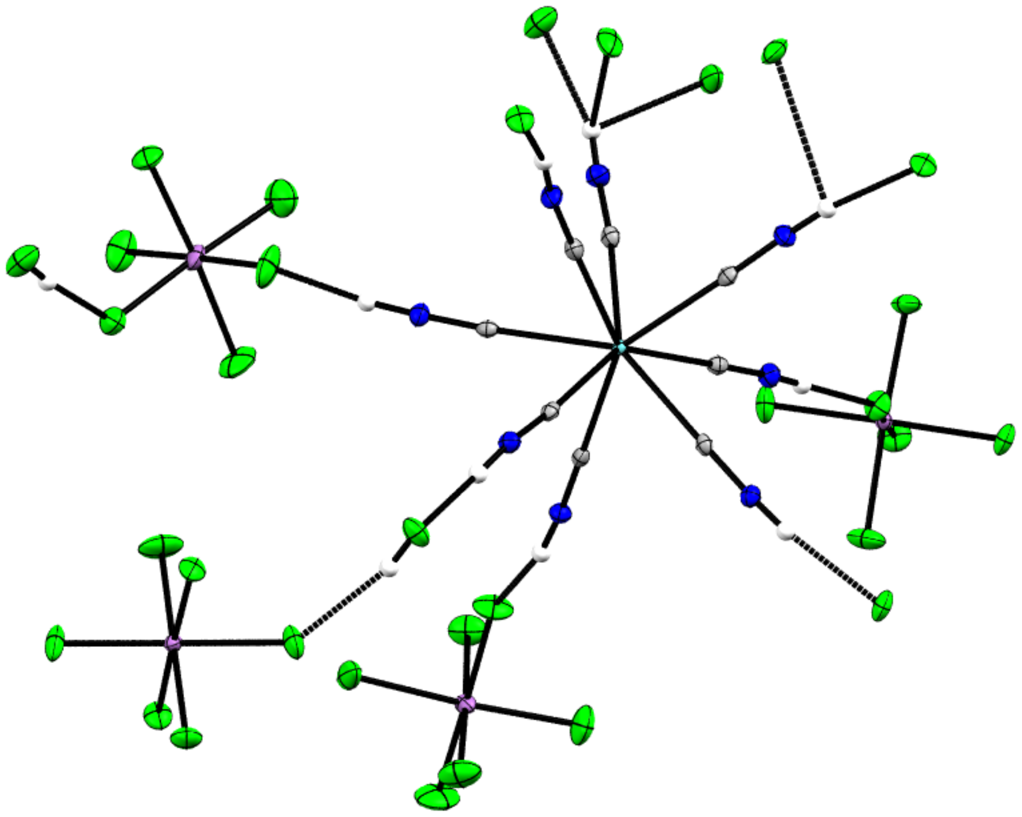

Figure S16. Network of H---F contacts in the crystal structure of $[Mo(CNH)_8]^{4+}$ $[SbF_6]^-_4$ • 2HF

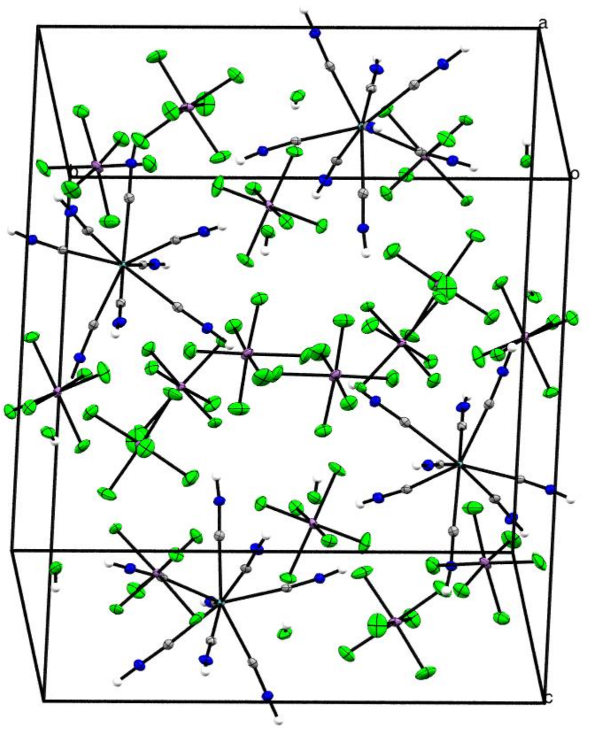

Figure S17. Unit cell of $[Mo(CNH)_8]^{4+}$ $[SbF_6]^-_4$ • 2HF

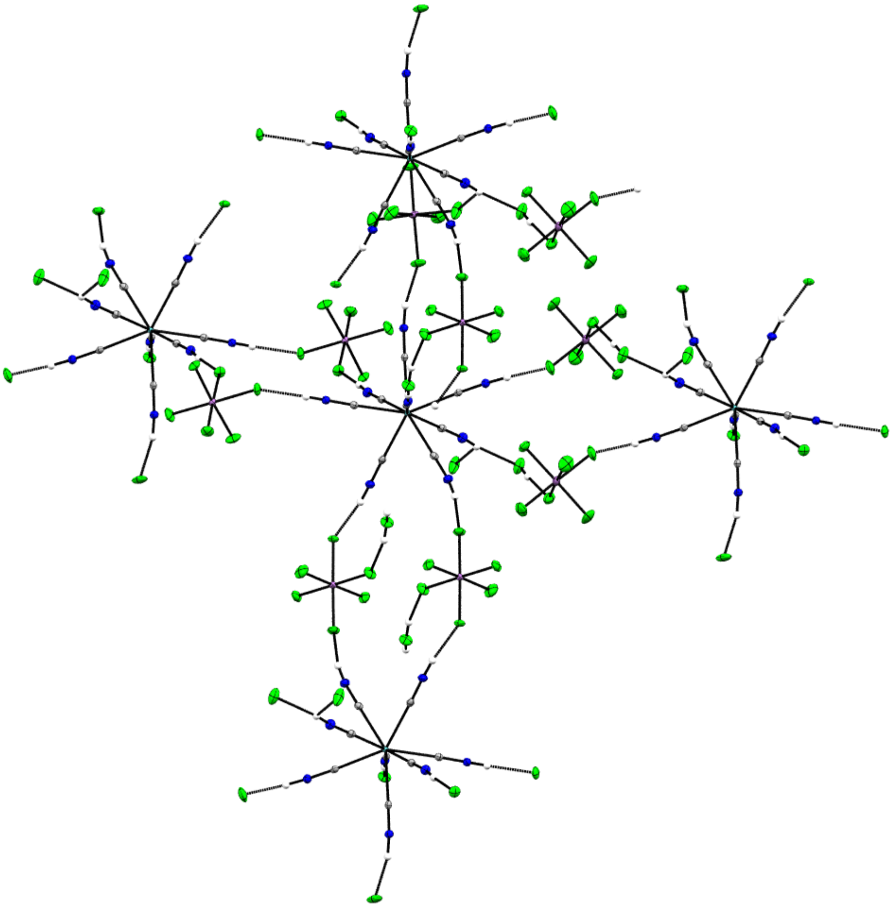

Figure S18. Hydrogen-bonded networks in $[Mo(CNH)_8]^{4+}$ $[SbF_6]^{-}_4$ • 2HF

Octakis(hydrogenisocyanide)molybdenum(IV) hexafluoroantimonate HF solvate

Crystal data

$MoC_8N_8H_8Sb_4F_{24}H_2F_2$	$F(000) = 2424$
$M_r = 1295.16$	$D_x = 2.866$ Mg m^{-3}
Monoclinic, $P2_1/n$	Mo $K\alpha$ radiation, $\lambda = 0.71073$ Å
$a = 10.9426$ (8) Å	Cell parameters from 9834 reflections
$b = 15.6430$ (11) Å	$\theta = 2.5$–$30.6°$
$c = 17.8663$ (13) Å	$\mu = 4.13$ mm^{-1}
$\beta = 91.087$ (3)°	$T = 100$ K
$V = 3057.7$ (4) Å^3	Block, yellow
$Z = 4$	0.43 × 0.32 × 0.22 mm

Data collection

Bruker D8 Venture diffractometer	8465 reflections with $I > 2\sigma(I)$
ϕ and ω scans	$R_{int} = 0.068$
Absorption correction: multi-scan	$\theta_{max} = 30.6°$, $\theta_{min} = 2.2°$
$T_{min} = 0.501$, $T_{max} = 0.746$	$h = -15 \rightarrow 15$
259625 measured reflections	$k = -22 \rightarrow 22$
9400 independent reflections	$l = -25 \rightarrow 25$

Refinement

Refinement on F^2	0 restraints
Least-squares matrix: full	Hydrogen site location: difference Fourier map
$R[F^2 > 2\sigma(F^2)] = 0.018$	All H-atom parameters refined
$wR(F^2) = 0.042$	$w = 1/[\sigma^2(F_o^2) + (0.0166P)^2 + 4.1529P]$ where $P = (F_o^2 + 2F_c^2)/3$
$S = 1.09$	$(\Delta/\sigma)_{max} = 0.004$
9400 reflections	$\Delta\rangle_{max} = 1.68$ e Å^{-3}
464 parameters	$\Delta\rangle_{min} = -1.37$ e Å^{-3}

The structure was refined with the expected Mo-CNH geometry as well as Mo-NCH:

Mo-CNH Isomer (carbon in grey, N in blue, H in white):

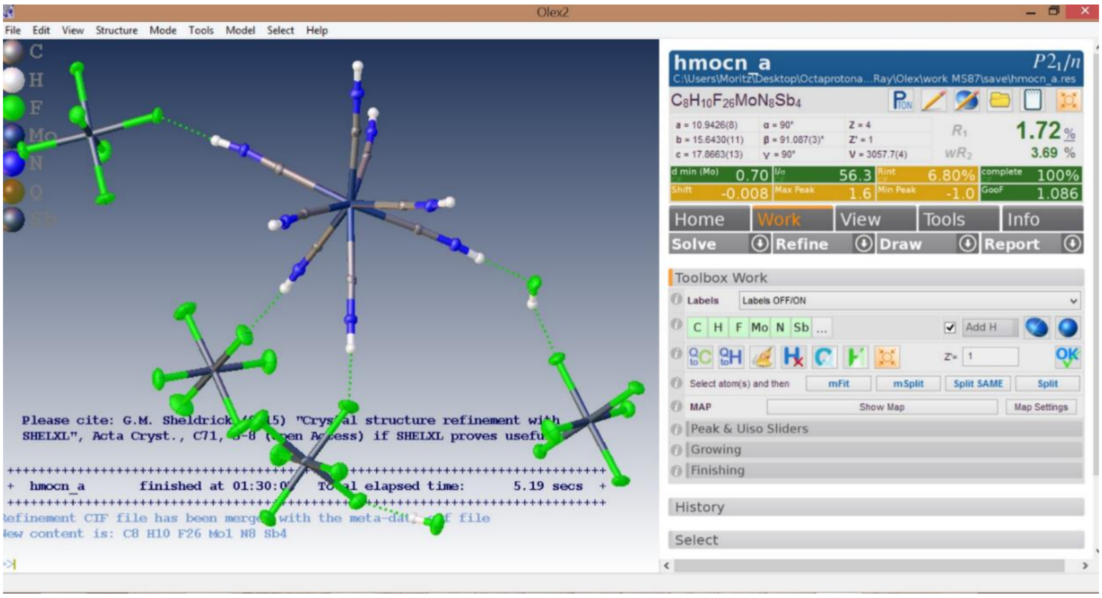

Figure S19. Structure refinement of $[Mo(CNH)_8]^{4+}$ isomer (carbon in grey, N in blue, H in white)

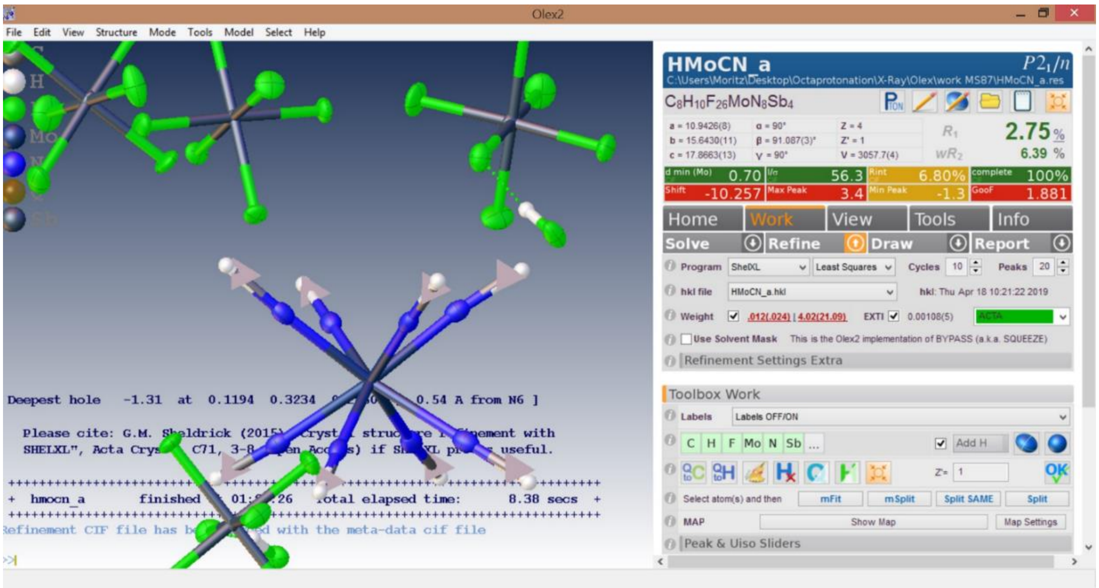

Figure S20. Structure refinement of $[Mo(NCH)_8]^{4+}$ isomer (carbon in grey, N in blue, H in white)

Geometric parameters (Å, º) for $[Mo(CNH)_8]^{4+}$ $[SbF_6]^-_4$ • 2HF

Sb01—F1	1.8586 (13)	Mo05—C1	2.143 (2)
Sb01—F2	1.8645 (13)	Mo05—C3	2.146 (2)
Sb01—F4	1.8649 (13)	Mo05—C6	2.152 (2)
Sb01—F3	1.8701 (13)	Mo05—C8	2.156 (2)
Sb01—F5	1.9025 (12)	Mo05—C4	2.161 (2)
Sb01—F6	1.9032 (12)	Mo05—C2	2.1617 (19)
Sb02—F7	1.8531 (14)	Mo05—C7	2.168 (2)

Sb02—F11	1.8552 (13)	F26—H9	0.72 (4)
Sb02—F8	1.8689 (13)	F25—H10	0.75 (4)
Sb02—F10	1.8864 (12)	N6—C6	1.130 (3)
Sb02—F9	1.8906 (13)	N6—H6	0.87 (4)
Sb02—F12	1.9027 (12)	N2—C2	1.128 (3)
Sb03—F24	1.8563 (15)	N2—H2	0.80 (4)
Sb03—F21	1.8599 (14)	N4—C4	1.131 (3)
Sb03—F23	1.8648 (15)	N4—H4	0.78 (4)
Sb03—F20	1.8704 (14)	N8—C8	1.136 (3)
Sb03—F22	1.8843 (14)	N8—H8	0.88 (3)
Sb03—F19	1.8975 (14)	N3—C3	1.129 (3)
Sb04—F18	1.8616 (13)	N3—H3	0.82 (4)
Sb04—F16	1.8650 (16)	N7—C7	1.131 (3)
Sb04—F13	1.8666 (15)	N7—H7	0.83 (4)
Sb04—F17	1.8728 (14)	N5—C5	1.131 (3)
Sb04—F14	1.8972 (14)	N5—H5	0.80 (4)
Sb04—F15	1.8986 (14)	N1—C1	1.134 (3)
Mo05—C5	2.140 (2)	N1—H1	0.87 (4)

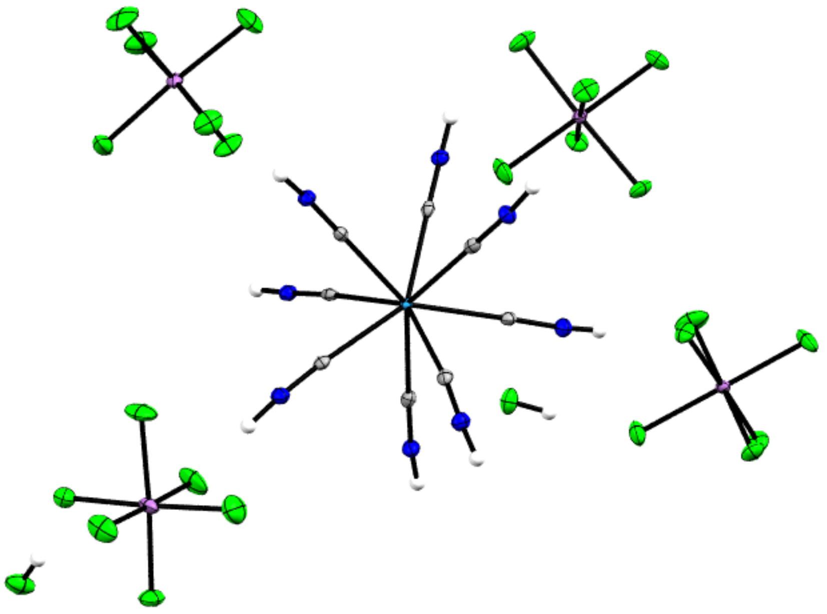

Figure S21: One formula unit in the crystal structure of $[W(CNH)_8]^{4+}$ $[SbF_6]^-{}_4$ • 2HF , ellipsoids shown at 50% probability, C grey, N blue, H white, W turquoise, F green, Sb lavender

Octakis(hydrogenisocyanide)tungsten(IV) hexafluoroantimonate HF solvate

Crystal data

$C_8H_8N_8W·4(F_6Sb)·2(FH)$	F(000) = 2504
M_r = 1383.09	D_x = 2.994 Mg m^{-3}
Monoclinic, $P2_1/n$	Mo $K\alpha$ radiation, λ = 0.71073 Å
a = 10.9494 (18) Å	Cell parameters from 9999 reflections
b = 15.681 (2) Å	θ = 2.3–31.1°
c = 17.871 (2) Å	μ = 7.39 mm^{-1}
β = 91.045 (5)°	T = 100 K
V = 3068.0 (8) Å^3	Block, clear yellow
Z = 4	0.24 × 0.23 × 0.11 mm

Data collection

Bruker D8 Venture diffractometer	R_{int} = 0.072
Absorption correction: multi-scan	θ_{max} = 31.1°, θ_{min} = 2.3°
T_{min} = 0.401, T_{max} = 0.746	h = -15→15
153354 measured reflections	k = -22→22
9841 independent reflections	l = -25→25
8997 reflections with $I > 2\sigma(I)$	

Refinement

Refinement on F^2	Hydrogen site location: difference Fourier map
Least-squares matrix: full	All H-atom parameters refined
$R[F^2 > 2\sigma(F^2)]$ = 0.018	$w = 1/[\sigma^2(F_o^2) + (0.0123P)^2 + 2.6019P]$ where $P = (F_o^2 + 2F_c^2)/3$
$wR(F^2)$ = 0.039	$(\Delta/\sigma)_{max}$ = 0.002
S = 1.10	$\Delta\rangle_{max}$ = 1.32 e Å^{-3}
9841 reflections	$\Delta\rangle_{min}$ = -1.18 e Å^{-3}
465 parameters	Extinction correction: *SHELXL2018*/1 (Sheldrick 2018), $Fc^*=kFc[1+0.001xFc^2\lambda^3/\sin(2\theta)]^{-1/4}$
0 restraints	Extinction coefficient: 0.00089 (3)
Primary atom site location: structure-invariant direct methods	

The structure was refined with the expected W-CNH geometry as well as W-NCH:

W-CNH Isomer (carbon in grey, N in blue, H in white):

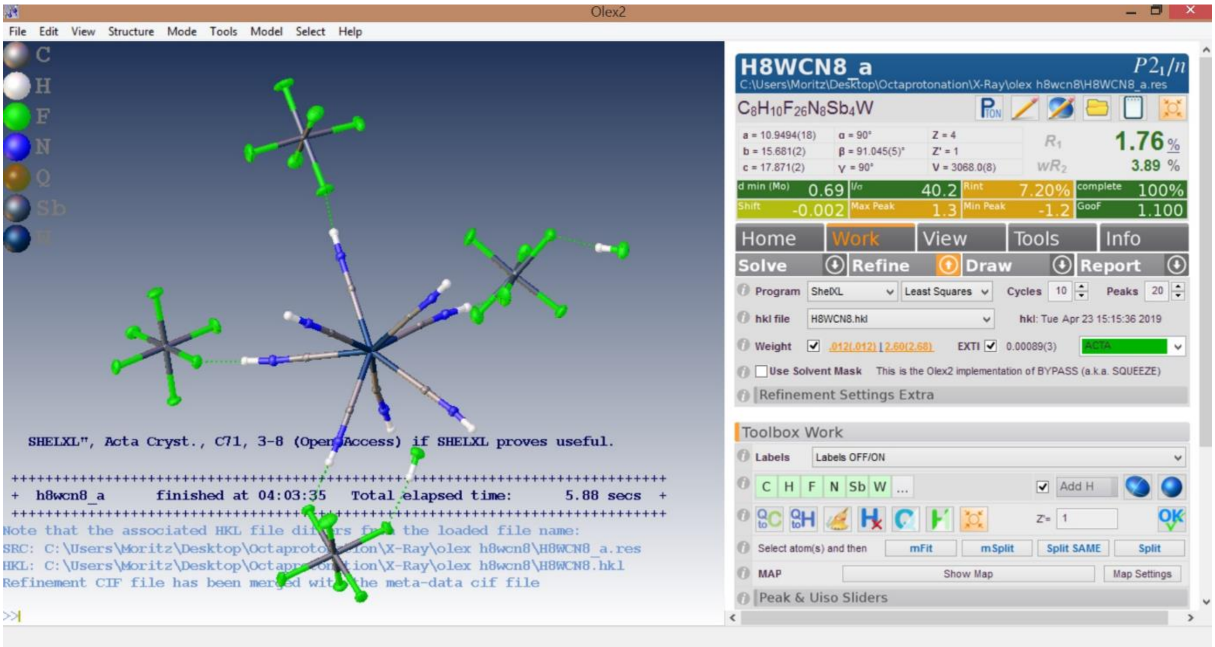

Figure S22. Structure refinement of $[W(CNH)_8]^{4+}$

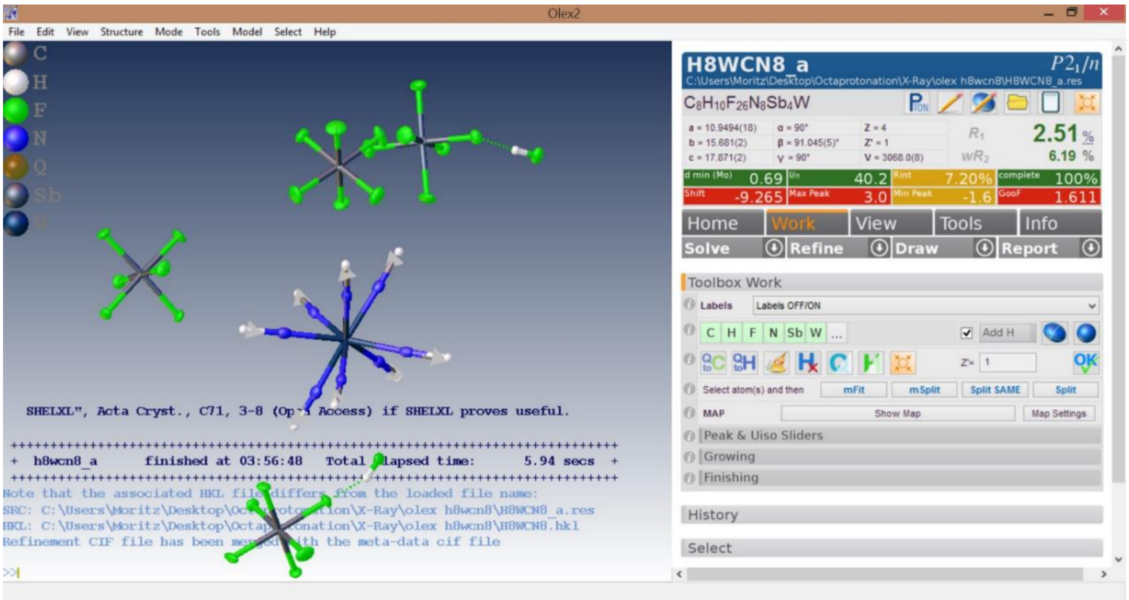

Figure S23. Structure refinement of $[W(NCH)_8]^{4+}$ (carbon in grey, N in blue, H in white):

Geometric parameters (Å, º) for $[W(CNH)_8]^{4+}$ $[SbF_6]^-_4$ • 2HF

W001—C5	2.142 (2)	Sb04—F00S	1.8594 (15)
W001—C2	2.145 (2)	Sb05—F00F	1.8661 (14)
W001—C1	2.157 (2)	Sb05—F00G	1.8997 (15)
W001—C7	2.169 (2)	Sb05—F00K	1.8795 (15)
W001—C6	2.149 (2)	Sb05—F00T	1.8701 (15)

W001—C4	2.167 (2)	Sb05—F00U	1.9022 (15)
W001—C8	2.166 (2)	Sb05—F00V	1.8636 (17)
W001—C3	2.163 (2)	F00E—H00E	0.81 (4)
Sb02—F006	1.8901 (13)	F00H—H00H	0.81 (4)
Sb02—F007	1.8603 (14)	N4—C4	1.129 (3)
Sb02—F009	1.9023 (13)	N4—H4	0.86 (4)
Sb02—F00A	1.8700 (14)	N8—C8	1.132 (3)
Sb02—F00M	1.8927 (14)	N8—H8	0.87 (4)
Sb02—F00O	1.8558 (15)	N7—C7	1.134 (3)
Sb03—F008	1.9046 (14)	N7—H7	0.94 (4)
Sb03—F00C	1.8620 (14)	N3—C3	1.127 (3)
Sb03—F00D	1.9043 (13)	N3—H3	0.91 (4)
Sb03—F00I	1.8686 (14)	N2—C2	1.135 (3)
Sb03—F00L	1.8671 (14)	N2—H2	0.77 (4)
Sb03—F00P	1.8706 (14)	C5—N5	1.137 (3)
Sb04—F00B	1.8856 (15)	N1—C1	1.137 (3)
Sb04—F00J	1.9010 (14)	N1—H1	0.87 (4)
Sb04—F00N	1.8626 (15)	N5—H5	0.88 (4)
Sb04—F00Q	1.8697 (15)	N6—C6	1.136 (3)
Sb04—F00R	1.8750 (15)	N6—H6	0.75 (4)

5. Continuous Shape-Measurements

Continuous-Shape Measurements were performed with the Programm Shape (v.2.1).[8]

For the MoC_8 fragment in $[Mo(CNH)_8]^{4+}$ $[SbF_6]^-_4$ • 2HF:

Ideal structures ML8

SAPR-8 5 D4d Square antiprism

TDD-8 6 D2d Triangular dodecahedron

JGBF-8 7 D2d Johnson gyrobifastigium J26

JETBPY-8 8 D3h Johnson elongated triangular bipyramid J14

JBTPR-8 9 C2v Biaugmented trigonal prism J50

BTPR-8 10 C2v Biaugmented trigonal prism

JSD-8 11 D2d Snub diphenoid J84

Structure 1 [H8Mo]

Mo	5.1065	2.4284	12.2447
C	5.9948	4.0999	11.2213
C	3.1999	1.4825	12.5214
C	3.9862	2.5422	10.4001
C	5.5995	0.4585	12.9860
C	7.0206	2.8251	13.1534
C	6.2851	1.4691	10.7335
C	4.7680	2.5451	14.3830
C	3.9013	4.1716	12.5404

SAPR-8 Ideal structure CShM = 0.37374

Mo	M	5.0958	2.4469	12.2427
C	L1	5.9917	4.1055	11.2101
C	L7	3.1901	1.5248	12.6138
C	L6	4.0302	2.5915	10.3817
C	L8	5.5575	0.4939	13.0121
C	L4	7.0717	2.6222	13.0701
C	L5	6.3976	1.5606	10.7800
C	L3	4.8037	2.5969	14.3668
C	L2	3.7237	4.0801	12.5068

TDD-8 Ideal structure CShM = 1.64179

Mo	M	5.0958	2.4469	12.2427
C	L1	6.1288	3.9059	11.0741
C	L7	3.3862	1.1825	12.4405
C	L3	3.8905	2.7932	10.5139
C	L6	5.8014	0.6099	13.0722
C	L5	6.8809	2.9199	13.3151
C	L2	6.0253	1.3835	10.6409
C	L8	4.5624	2.4789	14.3104
C	L4	4.0907	4.3018	12.5741

JGBF-8 Ideal structure CShM = 15.78434

Mo	M	5.0958	2.4469	12.2427
C	L1	5.5531	4.3512	11.0716
C	L5	3.5412	2.0428	12.0899
C	L2	4.4215	2.6407	10.0714
C	L7	6.1930	0.9469	13.5665
C	L3	6.6503	2.8511	12.3954
C	L6	5.5187	1.1407	11.3952
C	L8	4.2155	1.8490	14.2611
C	L4	4.6728	3.7532	13.0901

JETBPY-8 Ideal structure CShM = 28.65716

Mo	M	5.0958	2.4469	12.2427
C	L1	5.3304	3.9124	12.1267
C	L3	3.7772	2.9143	12.7516
C	L5	4.4271	2.6662	10.9308
C	L8	6.6340	0.5581	13.0491
C	L2	6.4988	2.4777	12.7393
C	L6	5.5955	1.2315	11.5434
C	L4	4.9456	1.4796	13.3642
C	L7	3.5575	4.3358	11.4362

JBTPR-8 Ideal structure CShM = 2.25289

Mo	M	5.0165	2.2105	12.0998
C	L7	6.0913	4.1906	10.8286
C	L5	3.2163	1.5755	12.6408
C	L3	4.0489	2.6378	10.4211
C	L6	5.5704	0.5493	13.0327
C	L2	6.6073	2.9312	13.0414
C	L4	6.4030	1.6116	10.8130
C	L8	4.6553	2.3585	14.6570
C	L1	4.2531	3.9574	12.6495

BTPR-8 Ideal structure CShM = 1.63290

Mo	M	5.0297	2.2502	12.1242
C	L7	5.9223	3.8947	11.0639
C	L5	3.0789	1.5631	12.7139
C	L3	3.9788	2.7108	10.3051
C	L6	5.6310	0.4510	13.1376
C	L2	6.7553	3.0328	13.1418
C	L4	6.5310	1.5987	10.7288
C	L8	4.7318	2.3764	14.2504
C	L1	4.2032	4.1448	12.7181

JSD-8 Ideal structure CShM = 4.27948

Mo	M	5.0958	2.4469	12.2427
C	L1	6.1212	4.0868	10.8062
C	L3	3.6650	1.4378	12.3285
C	L2	3.8584	2.9588	10.2399
C	L5	5.8292	0.4280	13.3335
C	L4	6.5821	2.8919	13.0586
C	L7	5.8769	1.5133	10.9812
C	L6	4.5743	2.3141	14.5909
C	L8	4.2591	3.9448	12.6022

For the WC_8 fragment in $[W(CNH)_8]^{4+}$ $[SbF_6]^-_4$ • 2HF:

Ideal structures ML8

SAPR-8 5 D4d Square antiprism

TDD-8 6 D2d Triangular dodecahedron

JGBF-8 7 D2d Johnson gyrobifastigium J26

JETBPY-8 8 D3h Johnson elongated triangular bipyramid J14

JBTPR-8 9 C2v Biaugmented trigonal prism J50

BTPR-8 10 C2v Biaugmented trigonal prism

JSD-8 11 D2d Snub diphenoid J84

Structure 1 [H8W]

W	5.2862	2.4335	3.3141
C	6.1791	4.1145	2.2863
C	5.7778	0.4592	4.0579
C	4.1622	2.5514	1.4624
C	3.3793	1.4824	3.5948
C	4.9513	2.5487	5.4534
C	7.2008	2.8306	4.2247
C	6.4673	1.4723	1.8030
C	4.0784	4.1780	3.6114

SAPR-8 Ideal structure CShM = 0.36936

W	M	5.2758	2.4523	3.3120
C	L1	6.1730	4.1146	2.2778
C	L7	5.7390	0.4952	4.0822
C	L5	4.2077	2.5976	1.4476
C	L8	3.3667	1.5278	3.6840
C	L3	4.9836	2.6018	5.4404
C	L2	7.2558	2.6280	4.1407
C	L6	6.5800	1.5650	1.8458
C	L4	3.9008	4.0884	3.5775

TDD-8 Ideal structure CShM = 1.64023

W	M	5.2758	2.4523	3.3120
C	L1	6.3103	3.9148	2.1415
C	L6	5.9836	0.6116	4.1426
C	L3	4.0679	2.7993	1.5801
C	L7	3.5633	1.1848	3.5100
C	L8	4.7416	2.4835	5.3838

C	L5	7.0644	2.9264	4.3866
C	L2	6.2074	1.3876	1.7067
C	L4	4.2682	4.3104	3.6448

JGBF-8 Ideal structure CShM = 15.77540

W	M	5.2758	2.4523	3.3120
C	L1	5.7336	4.3611	2.1396
C	L7	6.3757	0.9487	4.6376
C	L2	4.5999	2.6476	1.1365
C	L5	3.7182	2.0471	3.1588
C	L8	4.3941	1.8518	5.3343
C	L3	6.8335	2.8575	3.4652
C	L6	5.6998	1.1440	2.4621
C	L4	4.8519	3.7607	4.1619

JETBPY-8 Ideal structure CShM = 28.67483

W	M	5.2758	2.4523	3.3120
C	L1	5.5108	3.9205	3.1961
C	L8	6.8171	0.5598	4.1191
C	L3	4.6056	2.6723	1.9980
C	L5	3.9550	2.9204	3.8223
C	L6	5.1257	1.4829	4.4354
C	L2	6.6816	2.4830	3.8092
C	L4	5.7763	1.2348	2.6111
C	L7	3.7345	4.3448	2.5049

JBTPR-8 Ideal structure CShM = 2.23604

W	M	5.1964	2.2154	3.1687
C	L7	6.2726	4.2005	1.8957
C	L5	5.7521	0.5506	4.1026
C	L4	4.2264	2.6442	1.4871
C	L6	3.3928	1.5784	3.7109
C	L8	4.8349	2.3623	5.7313
C	L1	6.7904	2.9376	4.1123
C	L3	6.5856	1.6163	1.8788
C	L2	4.4312	3.9655	3.7206

BTPR-8 Ideal structure CShM = 1.62526

W	M	5.2097	2.2552	3.1932
C	L7	6.1035	3.9038	2.1315
C	L5	5.8128	0.4521	4.2077
C	L4	4.1563	2.7173	1.3709
C	L6	3.2552	1.5659	3.7841
C	L8	4.9114	2.3806	5.3238
C	L1	6.9386	3.0393	4.2130
C	L3	6.7139	1.6035	1.7945
C	L2	4.3811	4.1531	3.7894

JSD-8	Ideal structure	CShM =	4.24788	
W	M	5.2758	2.4523	3.3120
C	L1	6.3030	4.0965	1.8731
C	L5	6.0114	0.4288	4.4043
C	L2	4.0356	2.9658	1.3053
C	L3	3.8422	1.4406	3.3977
C	L6	4.7534	2.3181	5.6653
C	L4	6.7653	2.8982	4.1298
C	L7	6.0587	1.5174	2.0475
C	L8	4.4370	3.9530	3.6731

6. DFT-Calculations

Structure optimizations and frequency calculations were performed using Gaussian16[9] and M06L/Def2TZVP. Calculated frequencies were corrected by a scaling factor of 0.9965.[10]

XYZ-coordinates of optimized Structures in Å

$[Mo(CNH)_8]^{4+}$ E=-814.342 Hartree symmetry: C_1 but very close to D_{4d}

Mo-C 2.203 Å, C-N 1.146 Å, N-H 1.017 Å

Mo-C-N 179.9°, C-N-H 179.6°

C-Mo-C 72.4° (8x), 79.0° (8x), 113.3° (4x) 142.7° (8x)

Most intense IR frequencies: 689, 690, 696, 738, 741, 2241, 2245, 3554, 3556 cm^{-1}

42	0.000001000	-0.000002000	0.000028000
7	-2.253071000	-1.661508000	1.839011000
7	-1.661293000	2.253158000	1.839089000
7	-2.768250000	0.418085000	-1.838761000
7	0.418007000	2.767722000	-1.839528000
7	-0.418031000	-2.767793000	-1.839430000
7	2.768218000	-0.418141000	-1.838807000
7	1.661316000	-2.253072000	1.839172000
7	2.253082000	1.661557000	1.838954000
6	-1.481225000	-1.092543000	1.211299000
6	-1.092400000	1.481285000	1.211344000
6	-1.820123000	0.274738000	-1.211077000
6	0.274674000	1.819768000	-1.211577000
6	-0.274690000	-1.819821000	-1.211502000
6	1.820104000	-0.274775000	-1.211109000
6	1.092413000	-1.481235000	1.211393000
6	1.481237000	1.092580000	1.211252000
1	-0.544922000	-3.604547000	-2.402698000
1	2.934313000	2.163728000	2.402227000
1	-2.934354000	-2.163734000	2.402172000
1	0.545076000	3.604552000	-2.402639000
1	3.605278000	-0.545104000	-2.401602000
1	-3.605301000	0.545022000	-2.401575000
1	-2.163378000	2.934418000	2.402402000
1	2.163470000	-2.934288000	2.402477000

$[Mo(NCH)_8]^{4+}$ E=-814.386 Hartree

symmetry: C_1 but very close to D_{4d}

Mo-N 2.172 Å, N-C 1.145 Å , C-H 1.085 Å

Mo-N-C 179.9°, N-C-H 179.7°

N-Mo-N 73.3° (8x), 77.4° (8x), 115.2° (4x), 142.3° (8x)

Most intense IR frequencies: 429, 830, 833, 856, 859, 2207, 2209, 3271, 3273, 3274, 3280 cm^{-1}

42	0.000024000	0.000058000	0.000093000
7	1.739048000	-0.584443000	-1.162807000
7	0.584323000	1.738652000	-1.163428000
7	1.642638000	0.816686000	1.162965000
7	-0.816470000	1.642494000	1.163241000
7	0.816346000	-1.642412000	1.163319000
7	-1.642722000	-0.816612000	1.162780000
7	-0.584198000	-1.738549000	-1.163484000
7	-1.738916000	0.584507000	-1.162980000
6	2.654963000	-0.893350000	-1.777219000
6	0.893035000	2.654359000	-1.778249000
6	2.508420000	1.246299000	1.777517000
6	-1.246035000	2.508157000	1.777996000
6	1.245806000	-2.508170000	1.778015000
6	-2.508485000	-1.246318000	1.777293000
6	-0.892939000	-2.654351000	-1.778149000
6	-2.654775000	0.893264000	-1.777549000
1	1.183348000	3.519354000	-2.365741000
1	-1.183582000	-3.519752000	-2.364877000
1	-3.325949000	-1.651791000	2.364665000
1	3.326559000	1.650696000	2.364691000
1	1.650214000	-3.326341000	2.365135000
1	-3.520449000	1.183096000	-2.364279000
1	3.519777000	-1.184804000	-2.364416000
1	-1.651196000	3.325512000	2.365734000

$[Mo(CNH)_8]^{4+}$ • 8 HF   E=-1618.352 Hartree   Symmetry: C_1

Mo-C 2.188-2.189 Å, C-N 1.147 Å, N-H 1.049-1.050 Å, H···F 1.547-1.551 Å, H-F 0.930 Å

Mo-C-N 178.9-179.3°, C-N-H 177.5-177.9°, N-H···F 166.1-167.1°

C-Mo-C 72.4-72.9° (8x), 78.5-78.8° (8x), 113.5-114.0° (4x), 142.2-143.1° (8x)

Most intense IR frequencies: 403, 408, 416, 428, 888, 953, 962, 964, 972, 983, 987, 2196, 2200, 2988-3002, 3957-3959 cm^{-1}

42	0.000768000	-0.058754000	-0.005596000
9	-3.596017000	3.666451000	-2.818576000
9	-3.818110000	-3.295553000	-3.110014000
9	5.103897000	-0.232053000	2.936598000
9	-0.359576000	-5.238622000	2.782230000
9	3.242318000	3.850033000	-2.993021000
9	0.414677000	4.818280000	3.276078000
9	-4.969308000	0.435356000	3.116133000
9	3.968177000	-3.113767000	-3.106792000
7	-0.057610000	-2.880751000	1.772032000
7	-2.841744000	-0.058848000	1.740162000
7	-1.941705000	-1.997518000	-1.900806000
7	-1.937434000	1.955388000	-1.827436000
7	2.051945000	-1.997272000	-1.784913000
7	2.007979000	1.951283000	-1.754554000
7	2.756719000	-0.071643000	1.874544000
7	-0.031109000	2.716089000	1.846135000
6	-0.031900000	-1.904581000	1.169738000
6	-1.860442000	-0.069314000	1.145903000
6	-1.263107000	-1.333260000	-1.256871000
6	-1.267606000	1.256756000	-1.211540000
6	1.335678000	-1.336538000	-1.179424000
6	1.319641000	1.251507000	-1.160622000
6	1.802659000	-0.066814000	1.237398000
6	-0.027167000	1.758619000	1.214179000
1	-3.731735000	-0.004358000	2.293046000
1	3.649305000	-0.088293000	2.425962000
1	2.608606000	2.622399000	-2.293119000
1	-2.596126000	-2.585899000	-2.471496000
1	2.736244000	-2.565431000	-2.340910000
1	0.006115000	3.586468000	2.431320000
1	-0.104588000	-3.788877000	2.294679000
1	-2.565026000	2.607986000	-2.358313000
1	-3.919792000	4.034220000	-3.609558000
1	-4.004807000	-3.909236000	-3.783953000
1	5.591984000	-0.098838000	3.717452000
1	-0.178538000	-5.776325000	3.519667000
1	3.991259000	4.058926000	-3.504054000
1	0.011337000	5.520800000	3.733778000
1	-5.720949000	0.057198000	3.513373000
1	4.224117000	-3.869553000	-3.585293000

$[Mo(NCH)_8]^{4+}$ • 8 HF   E=-1618.359 Hartree   Symmetry: C_1

Mo-N 2.160-2.161 Å, N-C 1.146 Å , C-H 1.107 Å , H···F 1.666-1.668 Å, H-F 0.929 Å

Mo-N-C 178.5-178.7°, N-C-H 179.0-179.2° , C-H-F 164.1-164.6°

N-Mo-N 73.5° (8x), 77.2-77.3° (8x), 115.5-115.6° (4x), 142.2-142.3° (8x)

Most intense IR frequencies: 405, 455, 965, 2152-2158, 2966-2976, 3973-3977 cm^{-1}

42	0.011161000	0.003943000	-0.005103000
9	-0.060929000	5.344833000	-2.798756000
9	3.734092000	-3.833966000	2.775857000
9	-4.039023000	-3.419507000	2.855045000
9	-5.349042000	-0.242310000	-2.745118000
9	3.926348000	3.720542000	2.671837000
9	-3.737228000	3.918569000	2.625856000
9	5.405639000	-0.130998000	-2.686465000
9	-0.049420000	-5.421457000	-2.622514000
7	-1.288559000	-1.269687000	1.160505000
7	-1.282316000	1.314769000	1.126137000
7	-1.814105000	-0.007739000	-1.161788000
7	0.017663000	1.816657000	-1.181704000
7	0.010625000	-1.838750000	-1.133384000
7	1.843152000	-0.014501000	-1.150911000
7	1.297375000	-1.276215000	1.167999000
7	1.303199000	1.309245000	1.133927000
6	-1.996748000	-1.943508000	1.759139000
6	-1.977689000	2.021750000	1.701088000
6	-2.796649000	-0.022609000	-1.751976000
6	0.017701000	2.791700000	-1.784357000
6	0.008959000	-2.831731000	-1.706016000
6	2.830028000	-0.027945000	-1.733839000
6	1.988245000	-1.966128000	1.768546000
6	2.001702000	2.008666000	1.714281000
1	-0.019123000	5.943074000	-3.508628000
1	4.194858000	-4.221003000	3.483982000
1	-4.376980000	-3.986544000	3.509096000
1	-5.965173000	-0.162014000	-3.436126000
1	4.310219000	4.215370000	3.358382000
1	-4.201439000	4.330814000	3.317318000
1	6.020707000	-0.104899000	-3.382571000
1	-0.022532000	-6.042827000	-3.312984000
1	0.004782000	-3.799370000	-2.243720000
1	2.686874000	2.685157000	2.260417000
1	-2.695611000	-2.586888000	2.327606000
1	0.014029000	3.741664000	-2.352625000
1	3.791525000	-0.044063000	-2.282132000
1	-3.753429000	-0.045794000	-2.308425000
1	-2.652762000	2.713239000	2.241224000
1	2.658843000	-2.640519000	2.335207000

$[W(CNH)_8]^{4+}$ E=-813.253 Hartree

symmetry: C_1 but very close to D_{4d}

W-C 2.211 Å, C-N 1.147 Å, N-H 1.016 Å

W-C-N 179.9°, C-N-H 179.5-179.7°

C-W-C 72.4° (8x), 79.0° (8x), 113.3° (4x) 142.7° (8x)

Most intense IR frequencies: 679-681, 742-745, 2236-2239, 3554-3557 cm^{-1}

74	-0.000061000	-0.000064000	0.000108000
7	2.749704000	0.554201000	-1.845215000
7	-0.556813000	2.747574000	-1.847235000
7	1.554405000	2.337338000	1.842068000
7	-2.335140000	1.555450000	1.843800000
7	2.338545000	-1.551405000	1.843286000
7	-1.552749000	-2.334526000	1.847221000
7	0.554215000	-2.750994000	-1.843171000
7	-2.751834000	-0.557014000	-1.841200000
6	1.810205000	0.365163000	-1.215790000
6	-0.366946000	1.808618000	-1.217240000
6	1.022794000	1.539237000	1.213568000
6	-1.537569000	1.023437000	1.214954000
6	1.538986000	-1.021467000	1.215233000
6	-1.021795000	-1.537269000	1.217110000
6	0.365309000	-1.810957000	-1.214497000
6	-1.811444000	-0.367151000	-1.213339000
1	3.047294000	-2.016769000	2.403925000
1	-3.580701000	-0.724527000	-2.405160000
1	3.577691000	0.721217000	-2.410635000
1	-3.038268000	2.024438000	2.408436000
1	-2.021149000	-3.037708000	2.412300000
1	2.023145000	3.040606000	2.406762000
1	-0.724377000	3.575195000	-2.413019000
1	0.721277000	-3.579736000	-2.407466000

$[W(NCH)_8]^{4+}$ E=-813.302 Hartree

symmetry: C_1 but very close to D_{4d}

W-N 2.172-2.173 Å, N-C 1.146 Å , C-H 1.085 Å

W-N-C 179.9°, N-C-H 179.6°

N-W-N 73.3° (8x), 77.4-77.5° (8x), 115.2-115.3° (4x), 142.3° (8x)

Most intense IR frequencies: 407, 819-821, 861, 2200-2203, 3273 cm^{-1}

74	0.000006000	0.000012000	0.000065000
7	1.771562000	-0.477909000	-1.163069000
7	0.477632000	1.771103000	-1.163718000
7	1.590784000	0.914189000	1.163218000
7	-0.913947000	1.590508000	1.163647000
7	0.913870000	-1.590556000	1.163668000
7	-1.590800000	-0.914206000	1.163193000
7	-0.477665000	-1.771127000	-1.163702000
7	-1.771616000	0.477837000	-1.163074000
6	2.707065000	-0.729923000	-1.775184000
6	0.729675000	2.706394000	-1.776148000
6	2.430733000	1.397261000	1.775174000
6	-1.396836000	2.430369000	1.775873000
6	1.396811000	-2.430439000	1.775823000
6	-2.430780000	-1.397127000	1.775224000
6	-0.729489000	-2.706509000	-1.776082000
6	-2.707108000	0.729963000	-1.775155000
1	0.967464000	3.587792000	-2.362470000
1	-0.966937000	-3.588100000	-2.362250000
1	-3.222683000	-1.852683000	2.360467000
1	3.222425000	1.852953000	2.360598000
1	1.852480000	-3.222040000	2.361384000
1	-3.588819000	0.967830000	-2.360975000
1	3.588659000	-0.967567000	-2.361274000
1	-1.852239000	3.222147000	2.361401000

$[W(CNH)_8]^{4+}$ • 8 HF   E=-1617.263 Hartree   Symmetry: C_1

W-C 2.197-2.198 Å, C-N 1.148 Å, N-H 1.049 Å, H···F 1.548-1.552 Å, H-F 0.930 Å

W-C-N 179.0-179.3°, C-N-H 177.3-177.9°, N-H···F 166.3-167.1°

C-W-C 72.4-73.0° (8x), 78.5-79.0° (8x), 113.2-113.7° (4x), 142.5-143.1° (8x)

Intense IR frequencies: 413-418, 431, 880, 946, 972, 985, 2193-2197, 2991-3006, 3957-3959 cm^{-1}

74	0.003068000	-0.046565000	-0.004950000
9	-3.544820000	3.702868000	-2.867428000
9	-3.790274000	-3.260217000	-3.184621000
9	5.025106000	-0.342191000	3.085166000
9	-0.423747000	-5.265576000	2.725562000
9	3.278974000	3.845405000	-2.997908000
9	0.383204000	4.800336000	3.340926000
9	-4.951537000	0.424100000	3.161527000
9	3.968288000	-3.046171000	-3.180700000
7	-0.094726000	-2.901754000	1.736979000
7	-2.863740000	-0.049440000	1.718674000
7	-1.928966000	-1.965922000	-1.947303000
7	-1.899360000	1.990198000	-1.854805000
7	2.076381000	-1.971551000	-1.789553000
7	2.057004000	1.976763000	-1.701397000
7	2.721688000	-0.100712000	1.941581000
7	-0.048430000	2.725089000	1.868690000
6	-0.056157000	-1.918381000	1.146456000
6	-1.877969000	-0.058956000	1.130777000
6	-1.256158000	-1.310315000	-1.287853000
6	-1.243225000	1.286366000	-1.229234000
6	1.355298000	-1.317487000	-1.181574000
6	1.356382000	1.274938000	-1.123638000
6	1.783055000	-0.080367000	1.281265000
6	-0.038125000	1.772096000	1.229322000
1	-3.751225000	0.002044000	2.275665000
1	3.597826000	-0.135228000	2.517148000
1	2.663850000	2.644396000	-2.236808000
1	-2.576817000	-2.548083000	-2.531206000
1	2.760537000	-2.530055000	-2.354874000
1	-0.017841000	3.588773000	2.463945000
1	-0.152710000	-3.814139000	2.250567000
1	-2.515219000	2.647225000	-2.393005000
1	-3.847369000	4.084576000	-3.660149000
1	-3.973380000	-3.859508000	-3.872353000
1	5.505978000	-0.173716000	3.863570000
1	-0.263720000	-5.811632000	3.461712000
1	4.067116000	4.071402000	-3.437600000
1	-0.022064000	5.507787000	3.789241000
1	-5.740514000	0.059758000	3.493855000
1	4.254231000	-3.820874000	-3.609362000

$[W(NCH)_8]^{4+}$ • 8 HF   E=-1617.275 Hartree   Symmetry: C_1

W-N 2.161-2.163 Å, N-C 1.147 Å , C-H 1.107 Å, H···F 1.666-1.668 Å   H-F 0.929 Å

W-N-C 178.4-178.6°, N-C-H 179.0-179.2°, C-H-F 164.1-164.5°

N-W-N 73.4-73.5° (8x), 77.1-77.3° (8x), 115.4-115.6° (4x), 142.1-142.3° (8x)

Most intense IR frequencies: 425, 962, 2145-2152, 2960-2971, 3974-3976 cm^{-1}

74	-0.012943000	-0.007309000	-0.008259000
9	1.278129000	-5.180299000	-2.821020000
9	-4.459246000	2.957920000	2.780896000
9	3.223300000	4.142082000	2.929519000
9	5.192987000	1.358920000	-2.721947000
9	-3.073593000	-4.449957000	2.682192000
9	4.462057000	-3.061696000	2.635413000
9	-5.317272000	-0.952429000	-2.711129000
9	-1.069338000	5.319630000	-2.625649000
7	0.975585000	1.514964000	1.166026000
7	1.536041000	-1.008388000	1.120065000
7	1.767726000	0.407436000	-1.161487000
7	0.376511000	-1.772776000	-1.192856000
7	-0.412721000	1.797011000	-1.129752000
7	-1.805279000	-0.382507000	-1.157006000
7	-1.549580000	0.955534000	1.168341000
7	-0.988494000	-1.569095000	1.124303000
6	1.518806000	2.325334000	1.769088000
6	2.369468000	-1.549200000	1.693137000
6	2.726830000	0.636406000	-1.747309000
6	0.592580000	-2.723542000	-1.796884000
6	-0.624787000	2.769837000	-1.699065000
6	-2.772645000	-0.579426000	-1.740863000
6	-2.376425000	1.477315000	1.767959000
6	-1.519353000	-2.406152000	1.701359000
1	1.350849000	-5.778504000	-3.528376000
1	-5.014360000	3.216146000	3.479934000
1	3.413990000	4.797036000	3.560487000
1	5.805484000	1.440702000	-3.415941000
1	-3.315416000	-5.040382000	3.357778000
1	5.025531000	-3.338360000	3.320565000
1	-5.909623000	-1.145655000	-3.400527000
1	-1.259328000	5.918004000	-3.310733000
1	-0.826255000	3.716767000	-2.235113000
1	-2.043233000	-3.214180000	2.246764000
1	2.060172000	3.099758000	2.345237000
1	0.808358000	-3.647107000	-2.367258000
1	-3.714553000	-0.764443000	-2.291453000
1	3.658218000	0.864847000	-2.299759000
1	3.176814000	-2.079794000	2.233157000
1	-3.177458000	1.989801000	2.333980000

7. References

[1] J. G. Leipoldt, L. D. C. Bok, P. J. Cilliers, *Z. Anorg. Allg. Chem.* **1974**, *409*, 343-344.

[2] J. G. Leipoldt, L. D. C. Bok, P. J. Cilliers, *Z. Anorg. Allg. Chem.* **1974**, *407*, 350-352.

[3] G. Sheldrick, *Acta Crystallograph.* **2008**, *A64*, 112-122.

[4] Bruker, APEX2, SAINT, SADABS2014/15., *Bruker AXS Inc. Madison, Wisconsin, USA*.

[5] O. V. Dolomanov, L. J. Bourhis, R. J. Gildea, J. A. K. Howard, H. Puschmann, *J. Appl. Cryst.* **2009**, *42*, 339-341.

[6] C. F. Macrae, P. R. Edgington, P. McCabe, E. Pidcock, G. P. Shields, R. Taylor, M. Towler, J. van de Streek, *J. Appl. Cryst.* **2006**, *39*, 453-457.

[7] S. P. Westrip, *J. Appl. Cryst.* **2010**, *43*, 920-925.

[8] M. Llunell, D. Casanova, J. Cirera, J. M. Bofill, P. Alemany, S. Alvarez, SHAPE v. 2.1, **2013**.

[9] M. J. Frisch, G. W. Trucks, H. B. Schlegel, G. E. Scuseria, M. A. Robb, J. R. Cheeseman, G. Scalmani, V. Barone, G. A. Petersson, H. Nakatsuji, X. Li, M. Caricato, A. V. Marenich, J. Bloino, B. G. Janesko, R. Gomperts, B. Mennucci, H. P. Hratchian, J. V. Ortiz, A. F. Izmaylov, J. L. Sonnenberg, D. Williams-Young, F. Ding, F. Lipparini, F. Egidi, J. Goings, B. Peng, A. Petrone, T. Henderson, D. Ranasinghe, V. G. Zakrzewski, J. Gao, N. Rega, G. Zheng, W. Liang, M. Hada, M. Ehara, K. Toyota, R. Fukuda, J. Hasegawa, M. Ishida, T. Nakajima, Y. Honda, O. Kitao, H. Nakai, T. Vreven, K. Throssell, J. A. Montgomery, J. E. P. Jr., F. Ogliaro, M. J. Bearpark, J. J. Heyd, E. N. Brothers, K. N. Kudin, V. N. Staroverov, T. A. Keith, R. Kobayashi, J. Normand, K. Raghavachari, A. P. Rendell, J. C. Burant, S. S. Iyengar, J. Tomasi, M. Cossi, J. M. Millam, M. Klene, C. Adamo, R. Cammi, J. W. Ochterski, R. L. Martin, K. Morokuma, O. Farkas, J. B. Foresman, D. J. Fox, Gaussian, Inc., , Wallingford CT, **2016**.

[10] M. K. Kesharwani, B. Brauer, J. M. L. Martin, *J. Phys. Chem. A.* **2015**, *119*, 1701-1714.
